# Anti-Inflammatory Activity of Cajanin, an Isoflavonoid Derivative Isolated from *Canavalia lineata* Pods

**DOI:** 10.3390/ijms23169492

**Published:** 2022-08-22

**Authors:** Su-Jin Hong, Ok-Kyoung Kwon, Daseul Hwang, Su Hyun Goo, Doo-Young Kim, Min Ha Kim, Soo-Young Kim, Hyun-Jae Jang, Sei-Ryang Oh

**Affiliations:** 1 Natural Product Research Center, Korea Research Institute of Bioscience and Biotechnology, Cheonju 28116, Korea; 2 Natural Product Central Bank, Korea Research Institute of Bioscience and Biotechnology, Cheonju 28116, Korea; 3 National Institute of Biological Resources, Environmental Research Complex, Incheon 22689, Korea

**Keywords:** *Canavalia lineata*, isoflavonoid, cajanin, anti-inflammation

## Abstract

The bioactive components of *Canavalia lineata* (Thunb.) DC pods were investigated using bioactivity-guided isolation, and the chemical structures of flavonoids **1**–**3**, isoflavonoid derivatives **4**–**11**, and phenolic compounds **12** and **13** were identified by comparing NMR, MS, and CD spectral data with previously reported spectroscopic data. Compounds **1**–**13** were evaluated for their anti-inflammatory effects on LPS-stimulated RAW264.7 macrophages. Among these compounds, the isoflavonoid derivative cajanin (**7**) exhibited the most potent anti-inflammatory activity (IC_50_ of NO = 19.38 ± 0.05 µM; IC_50_ of IL-6 = 7.78 ± 0.04 µM; IC_50_ of TNF-α = 26.82 ± 0.11 µM), exerting its anti-inflammatory effects by suppressing the activation and nuclear translocation of the transcription factor NF-κB by phosphorylating IκB and p65. These results suggested that cajanin (**7**) may be a potential candidate for improving the treatment of inflammatory diseases.

## 1. Introduction

*Canavalia lineata* (Thunb.) DC. belongs to the genus *Canavalia*, which comprises approximately 51 species in the family Fabaceae, representing sword beans (*Canavalia gladiata*) and jack beans (*Canavalia ensiformis*) [1,2]. *Canavalia lineata* is a vine-type perennial plant that inhabits China, Japan, Taiwan, and the coastal region of Jeju Island in Korea. The mature beans are known to contain beneficial proteins and bioactive compounds [3], and the brown seeds grow to approximately 1.5 cm in pods 5–10 cm long and 3.0–3.5 cm wide. *Canavalia lineata* has been known to be a risk factor for fetal death in pregnant women and is traditionally used on Jeju Island to prevent pregnancy. The Korean name “Haenyeo-kong”, which means female diver bean, originates from its traditional use involving contraception [4,5].

The term homeostasis is usually defined as a physiologically balanced condition. In contrast, the term inflammation is often used to describe a response to an infection or injury caused by an extrinsic or intrinsic stimulus, which leads to an abnormal pathological condition due to a loss of homeostatic capacity [6]. Although the inflammatory response leads to a temporary disruption in homeostasis, the inflammatory process ultimately restores physiological homeostasis by eliminating pathogens such as microbes, fungi, and viruses [6,7]. However, dysregulated inflammation is the cause of inflammatory diseases, including allergies, asthma [8], rheumatoid arthritis [9], and sepsis [10]. Inflammatory signals are transferred indirectly or directly, depending on the particular cell type, to initiate or suppress certain core functions. For instance, macrophages contribute to a variety of roles, including wound healing, angiogenesis, tissue development, and vital innate immune responses to infectious pathogens [11,12]. However, the production of large amounts of inflammatory cytokines and mediators, such as nitric oxide (NO), tumor necrosis factor α (TNF-α), and interleukin-6 (IL-6), is induced by macrophage cells in response to adverse signs and can contribute to pathological inflammatory processes through signal transduction pathways, including the nuclear factor kappa-light-chain-enhancer of activated B cells (NF-κB) pathway [13,14]. Thus, restoring homeostasis from unregulated inflammatory processes by regulating these core pathways should be an effective approach for the treatment of inflammatory diseases.

*C. lineata* has been reported to be used not only as a female natural contraceptive, but also as livestock feed [4,5]; however, few studies have reported on the metabolites of *C. lineata*. Therefore, a phytochemical study on the composition of *C. lineata* may be applicable to the utilization of these natural resources as industrial crops. In the present study, the phytochemical components of the *C. lineata* pod methanol extract were purified by bioactivity-guided chromatographic separation to produce 13 compounds, and their chemical structures were elucidated based on NMR and MS spectroscopic data. Among these compounds **1**–**13**, cajanin (**7**), an isoflavonoid compound, exhibits a higher anti-inflammatory effect than that of other isolates by effectively inhibiting the inflammatory signaling mechanism and NF-κB pathways.

## 2. Results

### 2.1. Isolation and Identification of Compounds ***1***–***13*** from C. lineata Pods

**Figure 1:**
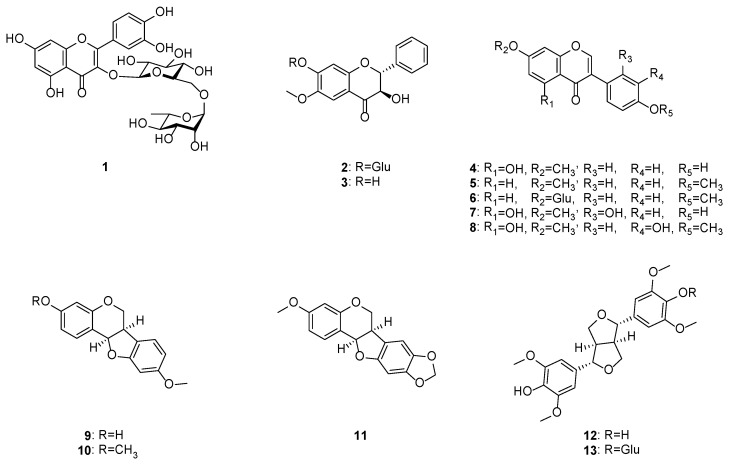
Chemical structures of compounds **1**–**13** obtained from *C. lineata* pods.

The methanol extract (152.1 g) of *C. lineata* pods suspended in H_2_O was successively partitioned into EtOAc- (15.2 g) and BuOH-soluble (9.6 g) fractions, which were subjected to C_18_ column chromatography, MPLC, and preparative HPLC to obtain 1 new flavonoid (**2**) and 12 known compounds (Figure 1 and Figure S1), including rutin (**1**) [15], (2*R*,3*R*)-3,7-dihydroxy- 6-methoxyflavanone (**3**) [16], prunetin (**4**) [17], 7,4′-dimethoxyisoflavone (**5**) [18], ononin (**6**) [19], cajanin (**7**) [20], 7,4′-dimethyl-3′-hydroxygenistein (**8**) [21], medicarpin (**9**) [19], homopterocarpin (**10**) [22], pterocarpin (**11**) [23], (+)-syringaresinol (**12**) [24], and (−)-syringaresinol-4-*O*-*β*-d-glucopyranoside (**13**) [24]. This is the first report of compounds **1**–**13** from *C. lineata*, and all the isolates were evaluated for their inhibitory activity on the LPS-stimulated production of the proinflammatory mediators NO, IL-6, and TNF-α in RAW264.7 cells.

### 2.2. Elucidation of (2R,3R)-3-Hydroxy 7-O-β-d-Glucopyranoside-6-Methoxyflavanone (Compound ***2***)

**Figure 2:**
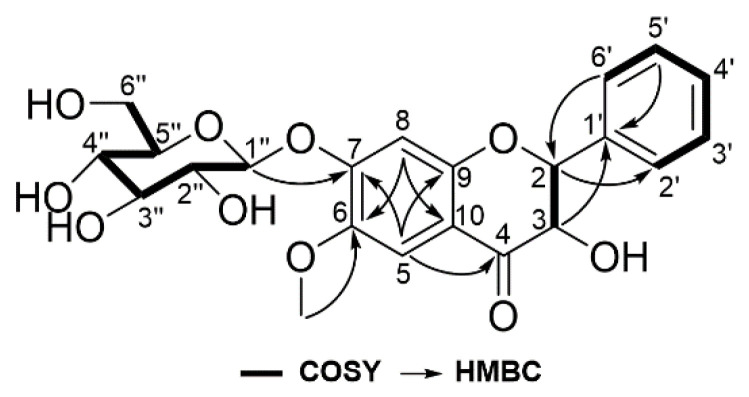
Key COSY and HMBC correlations of compound **2**.

Compound **2** was isolated as a yellow syrup with [*α*]$\begin{array}{c} 20\\ D \end{array}$ −50.8 (MeOH, *c* 0.1). Its molecular formula of C_22_H_24_O_10_ was determined from its HRESIMS data (*m*/*z* 447.1288 [M − H]^–^, calcd. 447.1291, Figure S2). The ^1^H and ^13^C NMR spectra (Table 1, Figures S3–S8) of compound **2** showed resonances that corresponded to an unsubstituted benzene ring [*δ*_H_ 7.55 (d, *J* = 7.0 Hz, H-2′, -6′), 7.42 (t, *J* = 7.0 Hz, H-3′, -5′), and 7.39 (t, *J* = 7.0 Hz, H-4′); *δ*_C_ 128.1 (C-2′, -6′), 128.2 (C-3′, -5′), 128.6 (C-4′), 137.5 (C-1′), B-ring], two singlet signals of a para-aromatic ring [*δ*_H_ 7.21, s, (H-5) and 6.77, s, (H-8); *δ*_C_ 107.2 (C-5), 103.3 (C-8), 112.3 (C-10), A-ring], three oxygenated aromatic carbons [*δ*_C_ 156.6 (C-9), 153.6 (C-7), 144.5 (C-6), A-ring], dihydroflavanol [*δ*_H_ 5.20 (d, *J* = 11.9 Hz, H-2) and 4.59 (d, *J* = 11.9 Hz, H-3); *δ*_C_ 83.9 (C-2), 72.6 (C-3)], one carbonyl carbon [*δ*_C_ 192.6 (C-4)], a sugar moiety bearing a *β*-anomeric proton [*δ*_H_ 5.05 (d, *J* = 7.7 Hz, H-1″), 3.25, (m, H-2″, -3″), 3.37, (m, H-4″), 3.14 (t, *J* = 9.1 Hz, H-5″), 3.63 (dd, *J* = 11.9, 9.1 Hz, H-6a″), 3.40 (dd, *J* = 11.9, 5.6 Hz, H-6b″); *δ*_C_ 99.4 (C-1″), 73.0 (C-2″), 76.6 (C-3″), 77.0 (C-4″), 69.4 (C-5″), 60.5 (C-6″)], and one methoxy group [*δ*_H_ 3.79, s; *δ*_C_ 55.9 (OCH_3_-6)]. The key COSY cross peaks between H-2/H-3 and H-4’/H-3′, -5′/H-2′, -6′ and the key HMBC cross peaks between H-2/C-2′, -6′, H-3/C-1′, H-5/C-4, -7, -9, H-8/C-6, -10, and OCH_3_/C-6 supported that the structure of compound **2** was similar to that of (2*R*,3*R*)-3,7′-dihydroxy-6-methoxyflavanone (**6**) except for a monosaccharide group (Figure 2). The linkage of the monosaccharide unit at C-7 of the aglycone was determined by the HMBC correlation between the anomeric proton at *δ*_H_ 5.05 (H-1″) and the oxygenated aromatic carbon at *δ*_C_ 153.6 (C-7). The absolute configuration at C-2 and C-3 of compound **2** were deduced as 2*R* and 3*R* on the basis of the observation of a positive and negative Cotton effect at 350 nm and 313 nm in the circular dichroism (CD) spectrum (Figure S9) [25].

The aglycone (**3**) and sugar of compound **2** were produced by the hydrolysis of compound **2** with 10% HCl, and the sugar derivative of compound **2** and d-, l-glucose and d-, l-galactose standards were analyzed using UPLC-QTOF-MS. The identity of the sugar of compound **2** was determined by comparing its retention time with those of the authentic sugar derivative peaks, which was verified by the mass fragmentation pattern of hexose derivatives, namely, *m*/*z* 455, 433, 320, 298, 262, 178, 160, and 136 [26]. The retention time (*t*_R_: 15.9 min) of the sugar derivative obtained from compound **2** corresponded to that of d-glucose (*t*_R_: 15.8 min, Figure S10). Therefore, the sugar of compound **2** was determined to be d-glucose, and the structure of compound **2** was elucidated as (2*R*,3*R*)-3-hydroxy 7-*O*-*β*-d-glucopyranoside-6-methoxyflavanone.

### 2.3. Inhibitory Effects of Compounds ***4*** and ***7*** on LPS-Enhanced Inflammatory Mediators

**Figure 3:**
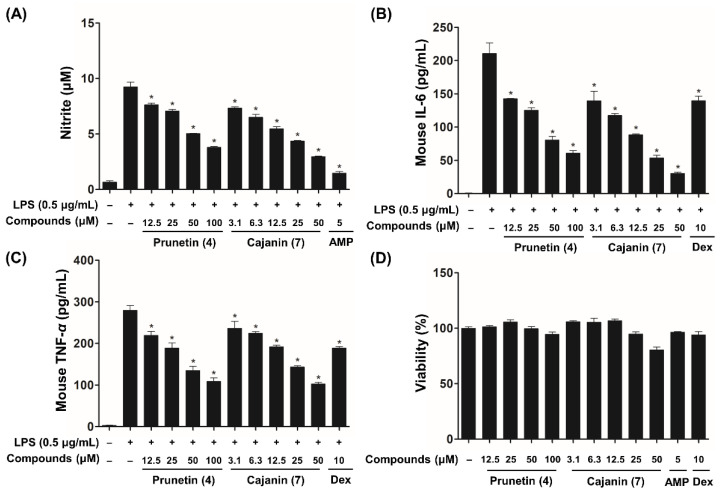
Effects of prunetin (**4**) and cajanin (**7**) on the inflammatory mediators NO (**A**), IL-6 (**B**), and TNF-*α* (**C**) in LPS-stimulated RAW264.7 macrophages. NO, IL-6, and TNF-*α* secretion was measured by NO and ELISAs as described in the Materials and Methods. Cytotoxicity was not observed at the IC_50_ concentrations of prunetin (**4**) and cajanin (**7**) (**D**), and all data are expressed as the mean ± SEM of three independent experiments. Statistical significance was considered at * *p* < 0.05 compared to the LPS-only treatment group. 2-Amino-4-methylpyridine (AMP) and dexamethasone (Dex) were employed as positive controls.

The anti-inflammatory effects of compounds **1**–**13** were tested using NO and ELISAs to evaluate the suppression of NO, IL-6, and TNF-α release. 2-Amino-4-methylpyridine (AMP) has been reported to inhibit the catalytic activity of the inducible NO synthases (NOS II) enzyme [27] and dexamethasone (Dex) is well known to reduce proinflammatory cytokines through the suppression of inflammatory pathways including the NF-κB signaling cascade [28]. Therefore, AMP (IC_50_ of NO = 5.67 ± 0.09 µM) and Dex (IC_50_ of IL-6 = 9.03 ± 0.14 µM; IC_50_ of TNF-*α* = 1.83 ± 0.25 µM) were used as positive controls in this screening assay due to their potent anti-inflammatory activity. After screening for the biologically active components, compounds **4**, **7**, and **9** exhibited a significant inhibitory effect on LPS-activated NO, IL-6, and TNF-α expression (Figures S15 and S16). Additionally, our bioassay results based on half maximal inhibitory concentration (IC_50_) values revealed that cajanin (**7**, IC_50_ of NO = 19.38 ± 0.05 µM; IC_50_ of IL-6 = 7.78 ± 0.04 µM; IC_50_ of TNF-*α* = 26.82 ± 0.11 µM) exhibited higher inhibitory activities than those of prunetin (**4**, IC_50_ of NO = 65.67 ± 0.09 µM; IC_50_ of IL-6 = 33.17 ± 0.08 µM; IC_50_ of TNF-α = 53.13 ± 0.07 µM) and medicarpin (**9**, IC_50_ of NO = 43.31 ± 0.06 µM; IC_50_ of IL-6 = 40.90 ± 0.08 µM; IC_50_ of TNF-α = >100 µM) (Figure 3 and Figure S17).

### 2.4. Anti-Inflammatory Effects of Compounds ***4*** and ***7*** through Inhibition of NF-κB Activation

**Figure 4:**
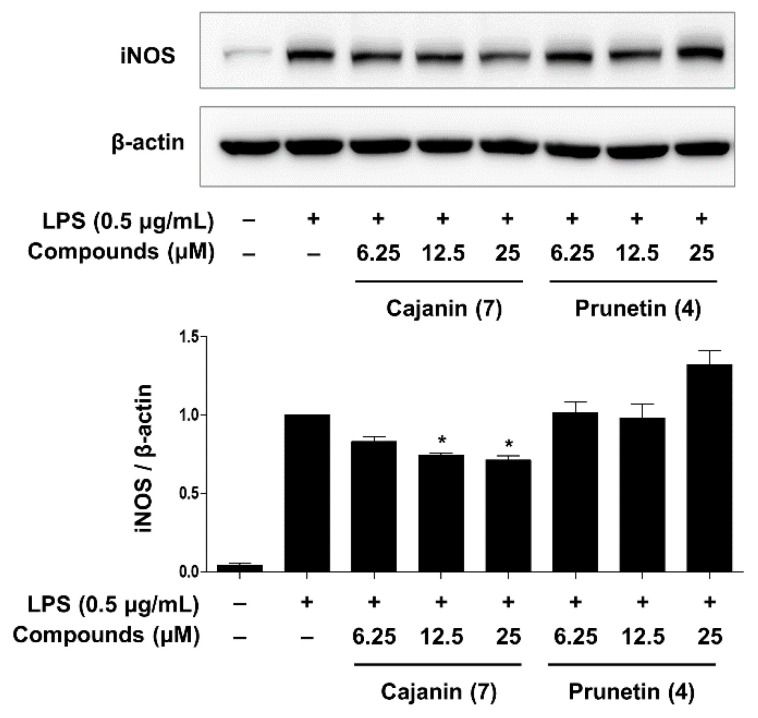
Effects of prunetin (**4**) and cajanin (**7**) on LPS-activated iNOS protein expression in macrophages. Western blot analysis was performed to measure iNOS protein levels normalized to that of *β*-actin as the loading control. The ratio of the iNOS/*β*-actin band intensities was calculated using the ImageJ program. The results are shown as the mean ± SEM of three independent experiments. Asterisks indicate a significant difference compared to the LPS alone group (* *p* < 0.05).

NO is catalyzed by the activation of nitric oxide synthase (NOS), which has the following isoforms: endothelial NOS (eNOS), neuronal NOS (nNOS), and inducible NOS (iNOS) [29]. eNOS and nNOS are constitutively expressed by endothelial cells in the vasculature and neurons in the nervous system, respectively, and relatively small amounts of NO are secreted by both eNOS and nNOS enzymes. However, extensive amounts of NO produced by iNOS, which is only activated in response to specific inflammatory signals, such as microbial or inflammatory mediators, are implicated in a variety of inflammatory diseases [30]. A Western blot analysis was performed to evaluate the inhibitory effects of prunetin (**4**) and cajanin (**7**) on iNOS protein expression in LPS-activated murine macrophages. As shown in Figure 4, cajanin (**7**) decreased the expression levels of iNOS in a dose-dependent manner, whereas prunetin (**4**) showed levels that were not significantly different from the comparison group treated with LPS alone. Therefore, compared to prunetin, cajanin (**7**) is a more effective than compound (**4**) for inhibiting the protein levels of iNOS that are stimulated by LPS-treated macrophages. These data were consistent with the inhibitory effect of prunetin (**4**) and cajanin (**7**) on NO production (Figure 3).

**Figure 5:**
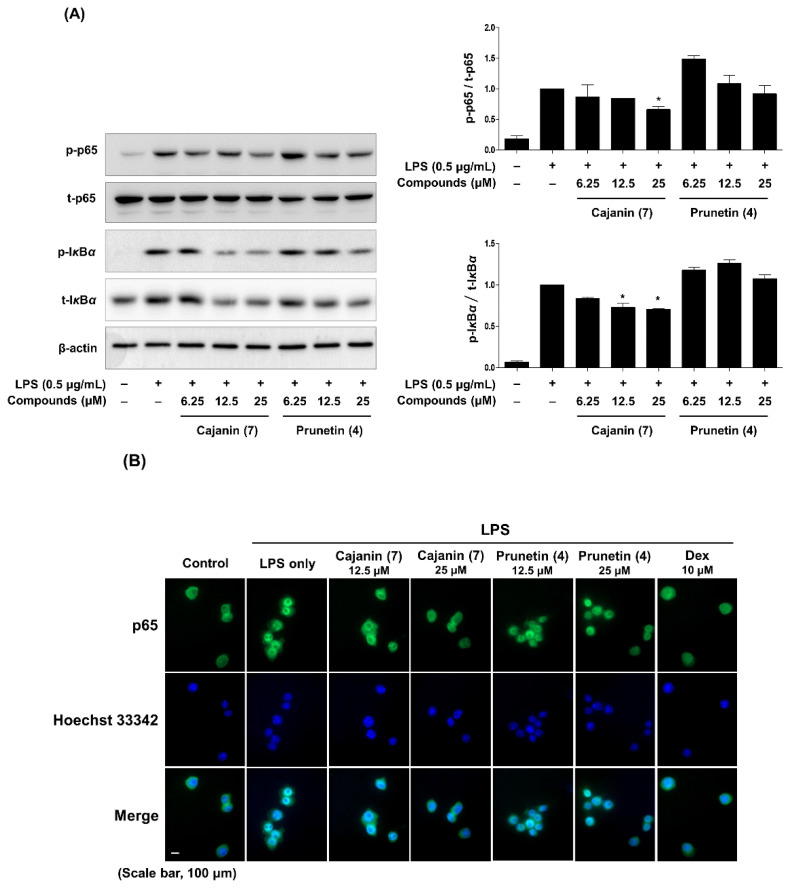
Effect of prunetin (**4**) and cajanin (**7**) on the phosphorylation of NF-κB (p65) and IκBα and nuclear translocation of NF-κB (p65) in LPS-induced macrophage cells. (**A**) Whole-cell lysates were prepared from RAW264.7 cells treated with or without LPS (0.5 μg/mL) and were subjected to Western blot analysis to investigate the phosphorylation of NF-κB (p65) and IκBα. The band intensities of phosphorylation of IκBα (p-IκBα) and NF-κB (p-p65) are represented as fold-change values using ImageJ software compared to total-IκBα (t-IκBα) and total-p65 (t-p65), which was used as a loading control. Data are indicated as the mean ± SEM of three independent experiments. Significant differences are expressed as asterisks between the LPS only and experimental groups (* *p* < 0.05). (**B**) Immunocytochemical analysis was performed to explore the nuclear localization of NF-κB (p65) using the fluorescence of Alexa Fluor 488 and Hoechst 33342. Dexamethasone (Dex) served as a positive control.

The inflammatory molecules iNOS, NO, TNF-α, and IL-6 are regulated by the transcription factor NF-κB, a key mediator of inflammatory responses [31]. To examine whether the activity of NF-κB was suppressed by cajanin (**7**) and prunetin (**4**), the phosphorylation of IκB (p-IκB) and p-p65 in whole-cell lysates was measured by Western blot analysis. As shown in Figure 5A, compared to prunetin (**4**), cajanin (**7**) markedly inhibited the expression of p-IκB and p-p65 at 12.5 and 25 μM compared to the group treated with LPS alone. In addition, an immunofluorescence analysis was performed to investigate the blockade effect of nuclear transcription factor (NF-κB) affected by cajanin (**7**) and prunetin (**4**). As shown in Figure 5B, cajanin (**7**) dramatically diminished the translocation of NF-κB (p65) to the nucleus at 25 μM, whereas prunetin (**4**) failed to completely block the translocation of NF-κB (p65) to the nucleus at this same concentration (Figure S18). These results suggested that the inhibitory effects of cajanin (**7**) on LPS-induced inflammatory mediators, such as NO, IL-6, TNF-α, and iNOS, were attributed to the blockade of NF-κB transcription factor activity via the suppression of p-p65 and p-IκB.

## 3. Discussion

Except for only a few bioactivities, such as antioxidant, anti-inflammatory, and antimelanogenesis effects, the biological activities of *C. lineata,* which naturally inhabits Jeju Island, Korea, have not been fully demonstrated [4,5]. Moreover, few studies have been conducted with the metabolites of *C. lineata*, with the exception of canavanine, one of the major nonproteinaceous amino acids of the legume family, which is isolated from *C. lineata* leaves and roots [32,33]. In the current study, the secondary metabolites of *C. lineata* pods were investigated by chromatographic purification and interpretation of spectroscopic data to generate 1 new flavonoid (**2**) and 12 known compounds, and their anti-inflammatory activities were explored on LPS-induced inflammatory molecules in RAW264.7 macrophages.

NO has a very short biological half-life in mammalian cells; however, an overproduction of NO is associated with many inflammatory diseases, such as asthma and rheumatoid arthritis. In the field of inflammation studies, this diatomic molecule has been used as a biomarker for bioactive plant extracts or compounds from many natural products [34]. Furthermore, proinflammatory cytokines, including IL-6 and TNF-*α*, are involved in inflammation-associated diseases and act as inflammatory mediators or activators of inflammatory pathways, such as NF-κB, mitogen-activated protein kinase (MAPK), and the Janus kinase (JAK)-signal transducer and activator of transcription (STAT) cascades [35].

In the search for bioactive metabolites that can reduce the inflammatory molecules that are enhanced by LPS stimulation, the methanol extract (1 g) of *C. lineata* pods was separated by an MPLC instrument equipped with a C_18_ column to yield 14 fractions (Figure S11). Of the fractions from which water-soluble substances were removed, MPLC fractions 8 and 9 showed an inhibitory effect against LPS-activated NO production without causing cytotoxicity in RAW264.7 cells. (Figures S12 and S13). Based on the UPLC chromatogram (280 nm) of MPLC fractions 8 and 9, in which compounds **3** (*t*_R_: 7.45 min), **7** (*t*_R_: 8.62 min), **9** (*t*_R_: 9.45 min), **4** (*t*_R_: 9.98 min), and **8** (*t*_R_: 10.18 min) were detected, it was inferred that flavonoid derivatives **3**, **4**, and **7**–**9** may contribute to the inhibitory effect of fractions 8 and 9 (Figure S14). As hypothesized for the biologically active components above, compounds **4**, **7**, and **9** exhibited a significant inhibitory effect on LPS-activated NO, IL-6, and TNF-α expression (Figures S15 and S16). From the NO bioassay and ELISA IC_50_ value results, cajanin (**7**) showed a 1.9- to 5.2-fold higher inhibitory activity than prunetin (**4**) and medicarpin (**9**) (Figure 3 and Figure S17). Based on the differences in the chemical structures of prunetin (**4**) and cajanin (**7**), the presence of the OH-2′ group in the B ring of cajanin may contribute to its anti-inflammatory activity. In previous studies, prunetin and medicarpin were reported to inhibit LPS-stimulated inflammatory mediator expression by suppressing the activation of the NF-κB pathway [36,37]; however, no studies have reported the anti-inflammatory effects of cajanin (**7**), which has shown a greater potency than prunetin (**4**) and medicarpin (**9**).

In the canonical NF-κB pathway, the NF-κB dimer, composed mainly of p65 (RelA) and p50 subunits, is inactivated by binding to the inhibitor of κB (IκB) proteins, including IκBα and IκBβ, and is sequestered in the cytoplasm. The IκB protein is phosphorylated by the multimeric IκB kinase complex (IKK) and is subsequently degraded by the 26S proteasome. Then, the released NF-κB dimer rapidly relocates from the cytoplasm into the nucleus, leading to the upregulation of the transcriptional activity of inflammatory genes, such as adhesion molecules, inflammatory cytokines, and chemokines [31]. The sustained activation of NF-κB is considered a powerful pathogenic factor that is involved in acute or chronic inflammatory disorders, including chronic obstructive pulmonary disease (COPD), asthma, multiple sclerosis, atherosclerosis, rheumatoid arthritis, and inflammatory bowel disease (IBD) [38]. Therefore, targeting NF-κB signal transduction cascades, including IKK kinase activity, proteasome activity, nuclear translocation, and DNA-binding activity, may be an effective therapeutic strategy for treating inflammatory diseases.

In this study, cajanin, an isoflavonoid isolated from *C. lineata* pods, significantly suppressed the phosphorylation and nuclear relocation of the transcription factor NF-κB, resulting in a reduction in the production of LPS-stimulated inflammatory molecules such as NO, IL-6, TNF-α, and iNOS. In addition to the NF-κB signaling pathway, our future studies will investigate the anti-inflammatory mechanism of cajanin in more detail.

## 4. Materials and Methods

### 4.1. General Experimental Procedures

NMR spectra [1D NMR (^1^H and ^13^C) and 2D NMR (COSY, HMQC, HMBC, and NOESY)] were recorded on JEOL ECZ-500R (JEOL, Tokyo, Japan) and Bruker Avance III HD 700 (Bruker, Billerica, MA, USA) instruments with methanol-*d*_4_ and DMSO-*d_6_* solvents. High-resolution electrospray ionization quadrupole time-of-flight mass spectrometry (HR-ESI-QTOF-MS) data were acquired using a Micromass Q-Tof Premier mass spectrometer (Waters, Milford, MA, USA) equipped with a UPLC-PDA system (Waters, Acquity UPLC system). Optical rotations were determined on a Jasco P-1000 polarimeter (Jasco Corp., Tokyo, Japan), and UV spectra were recorded using a SpectraMax M4 spectrophotometer (Molecular Devices, Sunnyvale, CA, USA). CD spectra were measured on a Jasco-J710 spectropolarimeter (Jasco Corp., Tokyo, Japan). The *C. lineata* pod MeOH extract was subjected to medium-pressure liquid chromatography (MPLC, Spot Prep II System, Armen, Paris, France) using a C_18_ column (YMC-ODS-AQ, 10 μm, 220 g, YMC, Tokyo, Japan). The preparative Gilson HPLC system (Gilson PLC 2020, Gilson, Middleton, WI, USA) and multiple preparative HPLC LC-Forte/R (YMC) using YMC-Triart C_18_ ExRS (20.0 mm × 250 mm, 10 μm, YMC) and YMC ODS-AQ (20.0 mm × 250 mm, 10 μm) columns were employed.

### 4.2. Plant Materials

*C. lineata* plants were collected in Gujwa-eup, Jeju-do, Republic of Korea, in September 2019. A voucher specimen (NIBRVP0000634114) of plant material was identified by one of the authors (M.-H. Kim) and was deposited at the National Institute of Biological Resources (NIBR). The plant was packaged and transported to the laboratory using refrigerating equipment and stored at −20 °C in Ziploc bags (Ziploc, S. C. Johnson & Son, Inc., Racine, WI, USA) for a maximum of 1 day.

### 4.3. Extraction and Isolation

The dried *C. lineata* pods (CLP, 1.2 kg) were extracted with MeOH (20 L × 3) solvents for 7 days at room temperature (RT). The MeOH extract was concentrated under reduced pressure to generate the residue (152 g). After suspending the crude extract in distilled water (1 L), it was successively partitioned with EtOAc (4 L × 3) and water-saturated BuOH (4 L × 3). The EtOAc-soluble fraction (15.2 g) was subjected to MPLC (Spot Prep II System, Armen, Paris, France) using a reversed-phase column eluted with a gradient of H_2_O:MeOH (YMC ODS-AQ, 10 μm, 220 g, 10–100% MeOH, 50 mL/min) to obtain 30 fractions (CLPE1–CLPE30) based on the MPLC chromatogram pattern. Compound **2** (6.0 mg) was purified from CLPE9 (63.8 mg) by preparative HPLC (YMC-Triart C_18_ ExRS, 20.0 mm × 250 mm, 10 μm, 10–33% ACN, 6 mL/min, *t*_R_ = 44.1 min). CLPE14 (91.6 mg) was separated chromatographically by preparative HPLC (YMC-Triart C_18_ ExRS, 5–40% ACN, 6 mL/min) to yield compounds **6** (2.6 mg, *t*_R_ = 44.3 min) and **12** (2.8 mg, *t*_R_ = 48.7 min). CLPE18 (65.7 mg) was isolated using preparative HPLC (YMC-Triart C_18_ ExRS, 5–35% ACN, 15 mL/min) to yield compound **3** (15.4 mg, *t*_R_ = 22.6 min), and CLPE21 (383.6 mg) was purified by preparative HPLC (YMC-Triart C_18_ ExRS, 5–55% ACN, 15 mL/min) to generate compound **7** (3.6 mg, *t*_R_ = 28.5 min). Additionally, preparative HPLC (YMC-Triart C_18_ ExRS, 10–50% ACN, 15 mL/min) was used to obtain compounds **9** (22.5 mg, *t*_R_ = 35.6 min) and **4** (3.3 mg, *t*_R_ = 30.7 min) from CLPE22 (222.3 mg) and CLPE24 (77.6 mg), respectively. CLPE27 (36.5 mg) was separated by preparative HPLC (YMC-Triart C_18_ ExRS, 25–43% ACN, 15 mL/min) to yield compound **8** (1.6 mg, *t*_R_ = 41.7 min), and CPLE28 (69.1 mg) was isolated by preparative HPLC (YMC-Triart C_18_ ExRS, 30–55% ACN, 15 mL/min) to yield compound **5** (2.8 mg, *t*_R_ = 33.9 min). CLPE30 (194.0 mg) was found to contain compounds **11** (2.7 mg, *t*_R_ = 29.1 min) and **3** (31.9 mg, *t*_R_ = 31.2 min) via preparative HPLC (YMC-Triart C_18_ ExRS, 30–60% ACN, 15 mL/min). The BuOH-soluble fraction (9.6 g) was chromatographed on a reversed-phase column (YMC ODS-AQ, 10 μm, 220 g) to yield CLPB1–CLPB25 using an MPLC instrument (5–100% MeOH, 50 mL/min). Additionally, preparative HPLC (YMC ODS-AQ, 20.0 × 250 mm, 10 μm, 20% ACN, 4 mL/min) was used to isolate compounds **13** (3.1 mg, *t*_R_ = 90.6 min) and **1** (8.0 mg, *t*_R_ = 62.7 min) from CLPB15 (31.2 mg) and CLPB16 (82.6 mg), respectively.

#### (2. R,3R)-3-Hydroxy 7-*O*-*β*-d-Glucopyranoside-6-Methoxyflavanone (2)

Yellow syrup; C_22_H_24_O_10_; [*α*]$\begin{array}{c} 20\\ D \end{array}$: −50.8 (*c* 0.1, MeOH); UV (MeOH) λ_max_ (logε) nm: 208 (2.3), 232 (2.1), 268 (1.8), 332 (1.7); CD (MeOH) λ_max_ (*Δ*ε) 214 (+3.9), 243 (+1.4), 313 (−3.1), 350 (+2.4) nm; ^1^H and ^13^C NMR spectra, see Table 1; HRESIMS: *m*/*z* 447.1288 [M–H]^–^ (calcd for C_22_H_24_O_10_, 447.1291).

### 4.4. UPLC-QTOF-MS Analysis for Determining the Sugar Content

In previous studies, the UPLC analysis method coupled with a QTOF-MS detector was employed to determine the absolute configuration of monosaccharide isomers, including hexose, glucose, galactose, allose and pentose, and xylose and arabinose [26]. Depending on our laboratory conditions, the method was modified to analyze the free sugar produced by acid hydrolysis of compound **2**. In short, compound **2** (2 mg) was dissolved in 70% EtOH that contained 10% HCl and was refluxed at 90 °C for 2 h. After acid hydrolysis was achieved, the reaction mixture was evaporated in vacuo. The sugar-containing aqueous layer was partitioned with an EtOAc layer and concentrated in a water bath. The dried residue was redissolved in anhydrous pyridine (1 mL, Sigma–Aldrich, St. Louis, MO, USA) and reacted with cysteine methyl ester (6 mg, TCI, Tokyo, Japan) at 60 °C for 1 h. Then, the reaction mixture was incubated with phenyl isothiocyanate (0.1 mL, Sigma–Aldrich) at 60 °C for 1 h, and the final product was analyzed by UPLC-QTOF-MS (Acquity UPLC system, Waters). The UPLC and ESI source conditions were configured according to a previously reported reference [26]. The d- and l-glucose and d- and l-galactose standards (1 mg each, TCI) were analyzed and prepared using the same protocol.

### 4.5. Cell Culture and Viability

RAW264.7 cells were purchased from American Type Culture Collection (ATCC No. TIB-71, Manassas, VA, USA) and cultured in Dulbecco’s modified Eagle’s medium (DMEM; Welgene, Daegu, South Korea) supplemented with penicillin–streptomycin (100 U/mL) and 10% heat-inactivated fetal bovine serum (FBS, Gibco, Grand Island, NY, USA) at 37 °C in a 5% CO_2_ humidified incubator. Cell viability was assayed using MTT (3-(4,5-dimethylthiazol-2-yl)-2,5-diphenyltetrazolium bromide) reagent, which is reduced by metabolically active cells. The cells were adhered at 1 × 10^4^ cells/well in 96-well plates and were treated with compound for 20 h. After further incubation for 4 h with MTT solution (0.25 mg/mL), the medium was aspirated, and the formazan was dissolved in DMSO. The optical density was measured at a wavelength of 570 nm (Spark 10 M, Tecan, Switzerland) and the cell viability was calculated by comparison with the control group [39]. Lipopolysaccharide (LPS from *Salmonella enterica* serotype typhimurium, L6511) and dexamethasone were purchased from Sigma–Aldrich, St. Louis, MO, USA.

### 4.6. Measurement of Nitric Oxide (NO) and Cytokine Production

The cells were plated at a density of 1 × 10^5^ cells/well in 96-well plates and were then stimulated with LPS (0.5 μg/mL) in various concentrations of compound for 20 h. The cell supernatants were collected for measurements of NO and cytokines, such as TNF-α and IL-6. NO was measured by the detection of its stable oxidative metabolite, nitrite. In brief, the cell supernatant (100 μL) was mixed with the same volume of Griess reagent. After reaction for 10 min at RT, the optical density was measured at 540 nm by a microplate reader (Spark10M, Tecan Science and Technology) and the nitrite concentration was determined using a sodium nitrite standard curve. The levels of TNF-α and IL-6 were measured using enzyme-linked immunosorbent assay (ELISA) kits (BD Biosciences, Santa Clara, CA, USA) according to the manufacturer’s protocols [40]. 2-Amino-4-methylpyridine (AMP) and dexamethasone (Dex) were used as positive controls.

### 4.7. Western Blot Analysis

Western blot analysis was performed to quantify the expression levels of protein in iNOS (1:1000; Enzo Life Science Inc., Farmingdale, NY, USA), p-I*κ*B-*α*, I*κ*B-*α* (1:1000; Invitrogen; Thermo Fisher Scientific, Waltham, MA, USA), p-p65, p65 (1:1000; Cell Signaling Technology, Beverly, MA, USA), and *β*-actin (1:3000; Santa Cruz Biotechnology, Santa Cruz, CA, USA) in RAW264.7 cells, as described in previous studies [40,41]. In brief, the cells were lysed by RIPA buffer (NP-40, Elpis Biotech, Daejeon, Korea) with protease inhibitors (Roche Molecular Systems, Branchburg, NJ) after pretreatment with compounds **4** and **7**. The purified proteins were analyzed by a BCA quantitative assay kit (Thermo Fisher Scientific, Waltham, MA, USA). An equivalent amount of each protein sample was loaded and separated by sodium dodecyl sulfate (SDS)-polyacrylamide gel electrophoresis (PAGE) and transferred onto polyvinylidene difluoride membranes (PVDF) (Millipore, Billerica, MA, USA). The membranes were then blocked with 5% skimmed milk and incubated with the primary antibodies at 4 °C overnight. Then, the membranes were incubated with horseradish peroxidase (HRP)-labeled secondary antibodies (1:5000; Jackson ImmunoResearch Laboratories, West Grove, PA, USA) for an additional 1 h at RT. The reaction complexes were visualized using an enhanced chemiluminescence reagent (Bio–Rad Laboratory, Hercules, CA, USA). The blot bands were quantified using ImageJ software (National Institute of Health, Bethesda, MD, USA).

### 4.8. Immunocytochemistry

Cells were adhered to 8-chamber slides, and the media was changed. After incubating with compounds **4** and **7** for 1 h and more, they were incubated with or without LPS (0.5 μg/mL). The cells were fixed with 4% formaldehyde and washed with phosphate-buffered saline (PBS). Then, the cells were permeabilized with Triton X-100 (0.2% in PBS) and incubated with primary antibody at 4 °C overnight. After washing, the membranes were incubated with a secondary antibody (Alexa Fluor 488, Thermo Fisher) for 1 h at RT. After further washing, the cells were incubated with Hoechst 33,342 to stain nuclei and measured under a fluorescence microscope (Axio Observer Z1, Carl Zeiss, Germany). The fluorescence intensity of p65 translocated to the nucleus of each cell was calculated by ImageJ software (ver. 1.53a, National Institutes of Health, Bethesda, MD, USA) and all fluorescence intensity values for each experimental group were normalized to the LPS-only treatment group.

### 4.9. Statistical Analyses

Data are indicated as the means ± standard deviation of the mean (S. D), and all experiments were carried out in triplicate. The statistical analysis was performed using Dunnett’s test in Prism 5 software (GraphPad software, San Diego, CA, USA). A *p* < 0.05 was considered statistically significant and is expressed as an asterisk.

## 5. Conclusions

In the current study, phytochemicals derived from *C. lineata* pods, a flavonol (**1**), flavanonols (**2** and **3**), isoflavonoid derivatives (**4**–**11**), and phenolic compounds (**12** and **13**), were obtained using bioactivity-guided isolation, and their chemical structures were identified by spectroscopic techniques, including NMR, QTOF-MS, and CD. To the best of our knowledge, the present study is the first to report that compounds **1**–**13** are phytochemical components of *C. lineata* pods. All isolates were screened for their inhibitory effect on LPS-enhanced inflammatory mediators, such as NO, IL-6, and TNF-α, in macrophages. Among compounds **1**–**13**, isoflavonoid derivatives prunetin (**4**) and cajanin (**7**) exhibited potent anti-inflammatory activity, and cajanin (**7**) proved to be an effective bioactive component that compared to prunetin more greatly inhibits NF-κB activity (**4**). These results suggested that differences in the chemical structure of the B ring of isoflavonoids may be associated with anti-inflammatory effects. Therefore, cajanin (**7**), which is elicited from an unused natural resource, *C. lineata* pods, could be a useful candidate for the treatment of inflammatory diseases.

## Figures and Tables

**Table 1:** ^1^H (700 MHz) and ^13^C NMR (175 MHz) data of **2**^a^.

Position	2 ^a^		HMBC
	*δ*_H_ (*J* in Hz)	*δ*_C_	H→C
2	5.20, d (11.9)	83.9	3, 4, 1′, 2′, 6′
3	4.59, d (11.9)	72.6	2, 4, 1′
4	-	192.6	-
5	7.21, s	107.2	4, 6, 7, 9, 10
6	-	144.5	-
7	-	153.6	-
8	6.77, s	103.3	4, 6, 7, 9, 10
9	-	156.6	-
10	-	112.3	-
1′	-	137.5	-
2′, 6′	7.55, d (7.0)	128.1	2, 2′, 4′, 6′
3′, 5′	7.42, t (7.0) ^b^	128.2	1′, 3′, 5′
4′	7.39, t (7.0) ^b^	128.6	2′, 6′
1″	5.05, d (7.7)	99.4	7, 5″
2″	3.25 m ^b^	73.0	1″, 2″, 3″
3″	3.25 m ^b^	76.6	1″, 2″, 3″
4″	3.37, m ^b^	77.0	5″, 6″
5″	3.14, t (9.1)	69.4	3″, 4″, 6″
6″	3.63, dd (11.9, 9.1)	60.5	4″, 5″
	3.40, dd (11.9, 5.6)		
OCH_3_-6	3.79, s	55.9	6

^a^ Data were recorded in DMSO-*d*_6._^b^ Overlapping.

## Data Availability

Not applicable.

## Supplementary Materials

The supporting information can be downloaded at: https://www.mdpi.com/article/10.3390/ijms23169492/s1.
