# Supplementary material for: Anti-Inflammatory Activity of Cajanin, an Isoflavonoid Derivative Isolated from Canavalia lineata Pods
Source: Int J Mol Sci. 2022 Aug 22;23(16):9492. doi: 10.3390/ijms23169492 (PMC9409483; doi:10.3390/ijms23169492)
Supplement: Supplementary file 1 [file ijms-23-09492-s001.zip › ijms-1840195-supplementary.pdf]

Supplementary Materials

Anti-inflammatory activity of cajanin, an isoflavonoid derivative isolated from *Canavalia lineata* pods

Su-Jin Hong[1,†], Ok-Kyoung Kwon[1,2,†], Daseul Hwang[1], Su Hyun Goo[1], Doo-Young Kim[1,2], Min-Ha Kim[3], Soo-Young Kim[3], Hyun-Jae Jang[1,2,*]and Sei-Ryang Oh[1,2,*]

[1]Natural Product Research Center, Korea Research Institute of Bioscience and Biotechnology, Cheonju, 28116, Republic of Korea

[2]Natural Product Central Bank, Korea Research Institute of Bioscience and Biotechnology, Cheonju, 28116, Republic of K orea

[3]National Institute of Biological Resources, Environmental Research Complex, Incheon, 22689, Republic of Korea

[†] These authors contributed equally to this work.

*Corresponding author: E-mail address: water815@kribb.re.kr (H.-J. Jang); seiryang@kribb.re.kr (S.-R. Oh)

Content		Page
**Figure S1.**	Scheme for isolation of phytochemicals **1–13** from CLP	S1
**Figure S2.**	HR-ESI-QTOF-MS spectrum of **2**	S2
**Figure S3**	^{1}H NMR (700 MHz, DMSO-d_6) spectrum of **2**	S3
**Figure S4**	^{13}C NMR (175 MHz, DMSO-d_6) spectrum of **2**	S4
**Figure S5**	COSY (700 MHz, DMSO-d_6) spectrum of **2**	S5
**Figure S6**	HMQC (700 MHz, DMSO-d_6) spectrum of **2**	S6
**Figure S7**	HMBC (700 MHz, DMSO-d_6) spectrum of **2**	S7
**Figure S8**	NOESY (700 MHz, DMSO-d_6) spectrum of **2**	S8
**Figure S9.**	CD spectrum of **2** and **3**	S9
**Figure S10.**	Determination of sugar contents using UPLC-QTOF-MS	S10
**Figure S11.**	MPLC fractionation of *Canavalia lineata* pod (CLP) MeOH extract for bioactivity-guided separation.	S11
**Figure S12.**	Anti-inflammatory effects of CLP MeOH fractions 1–14	S12
**Figure S13.**	Anti-inflammatory effects of bioactive subfractions 8, 9, and 11	S13
**Figure S14.**	UPLC analysis (280 nm) of bioactive subfractions 8–11	S14
**Figure S15.**	Anti-inflammatory effects of compounds **1**–**13** on LPS-induced NO production.	S15
**Figure S16.**	Anti-inflammatory effects of compounds **1**–**13** on LPS-induced IL-6 and TNF-α production.	S16
**Figure S17.**	Anti-inflammatory effects of compounds **4**, **9,** and **7** on LPS-induced NO, IL-6, and TNF-α production.	S17
**Figure S18**	Fluorescence intensity of p65 translocated to the nucleus.	S18
**Table S1.**	Climate condition for harvesting of *C. lineata*	S19

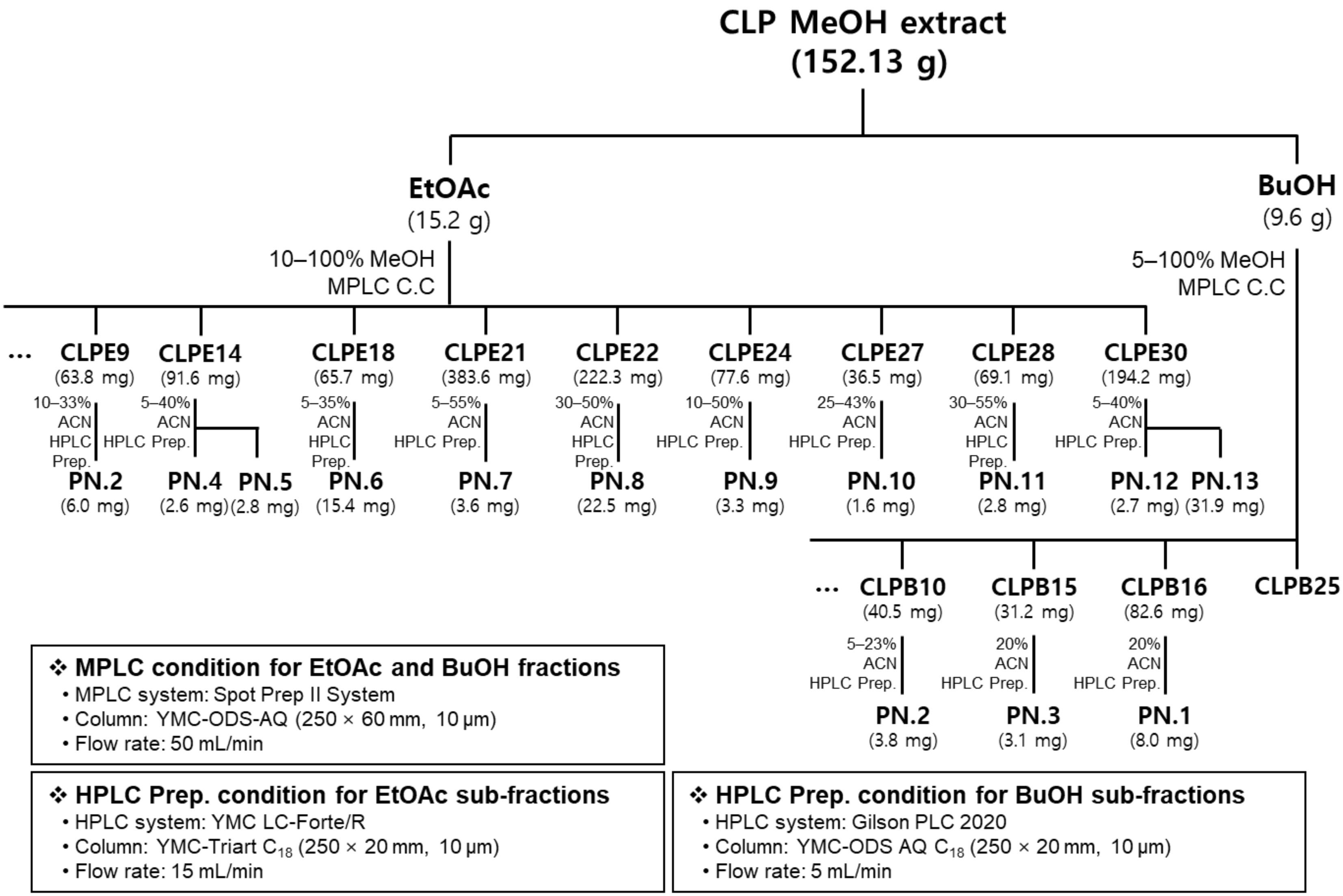

Figure S1. Scheme for isolation of phytochemicals **1**–**13** from CLP.

Mass	Calc. Mass	mDa	PPM	DBE	Formula	C	H	O
447.1288	447.1291	-0.3	-0.7	11.5	C22 H23 O10	22	23	10

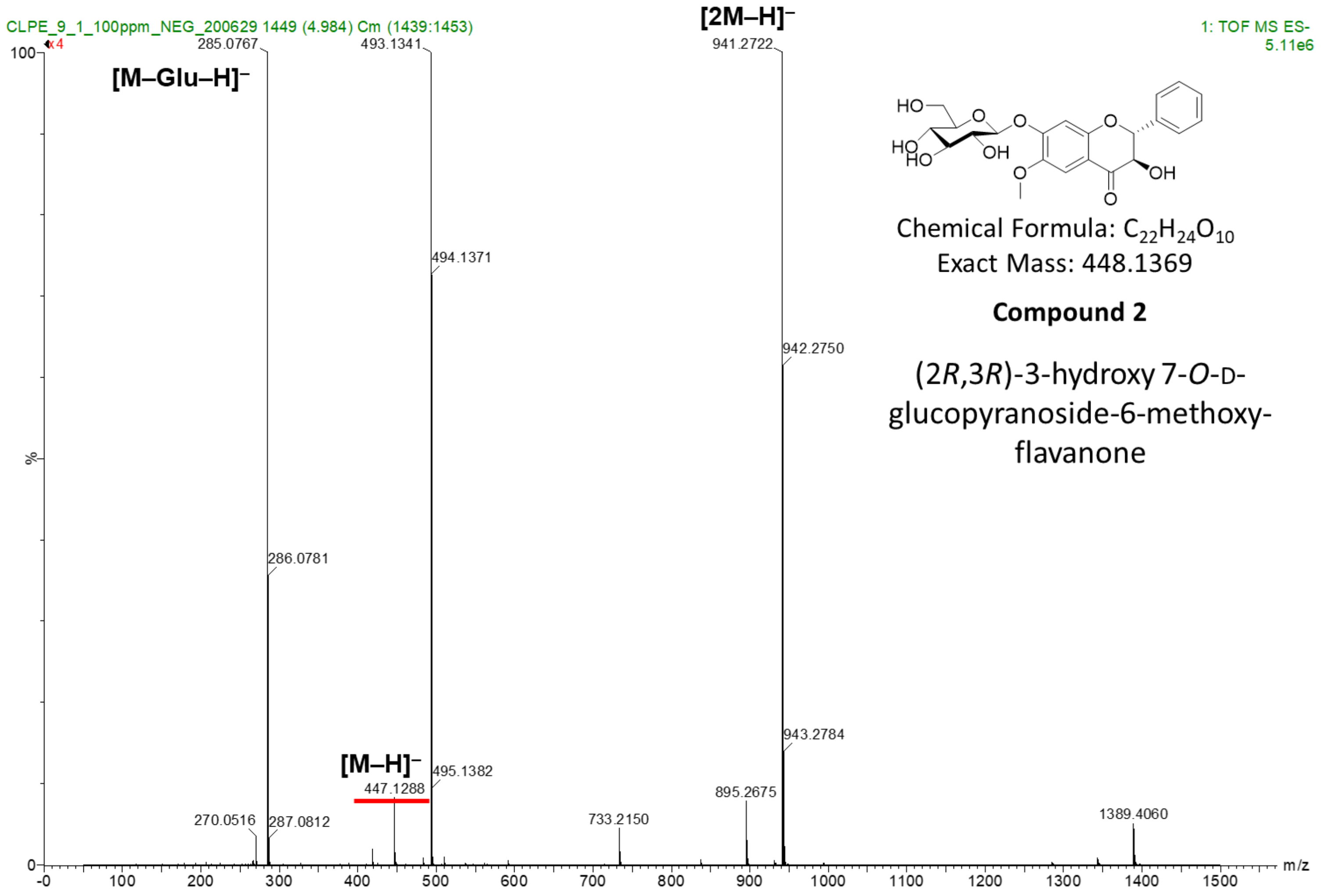

Figure S2. HR-ESI-QTOF-MS spectrum of **2.**

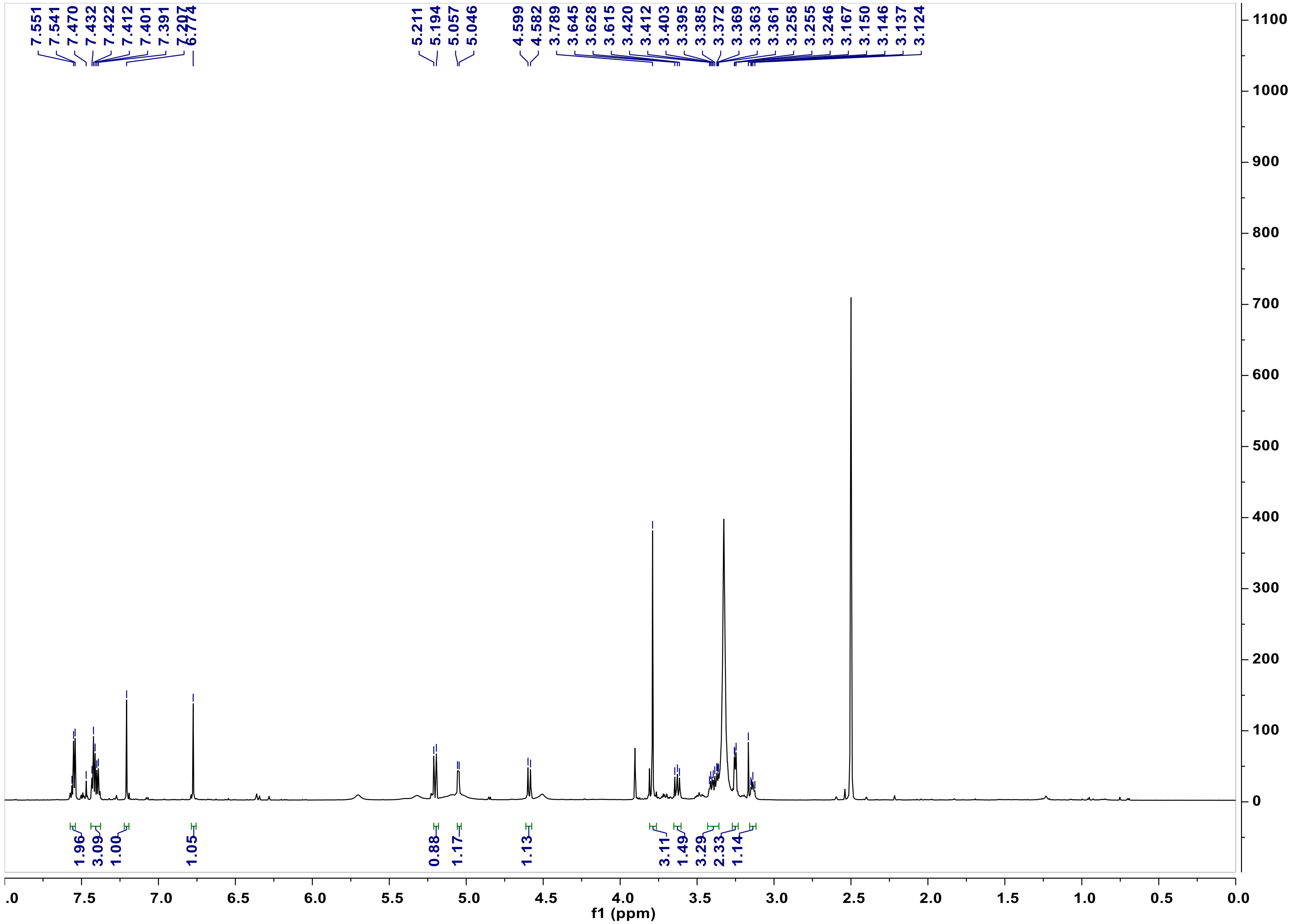

Figure S3. ^{1}H NMR (700 MHz, DMSO-d_6) spectrum of **2.**

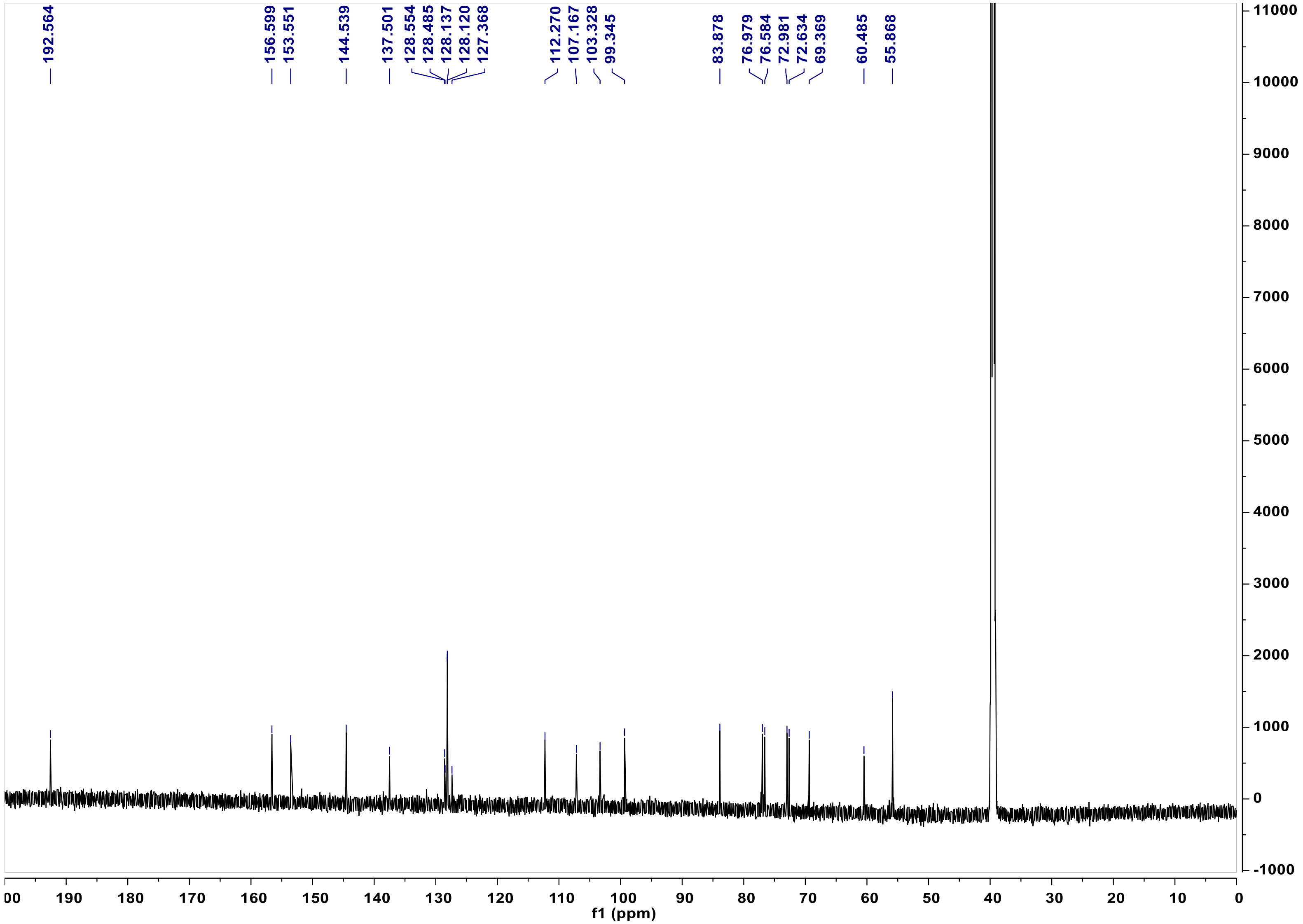

Figure S4. ^{13}C NMR (175 MHz, DMSO-d_6) spectrum of **2.**

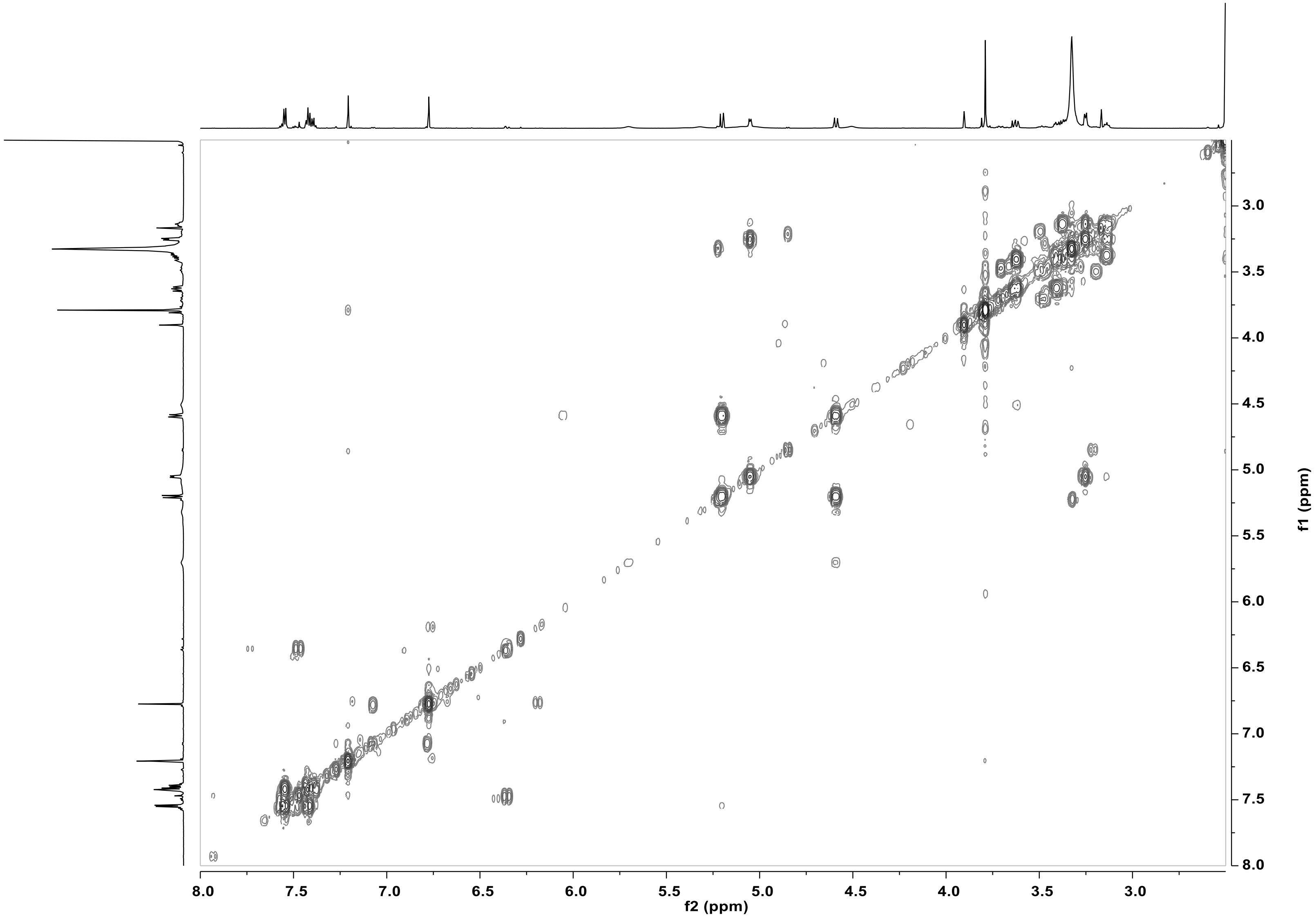

Figure S5. COSY (700 MHz, DMSO-d_6) spectrum of **2.**

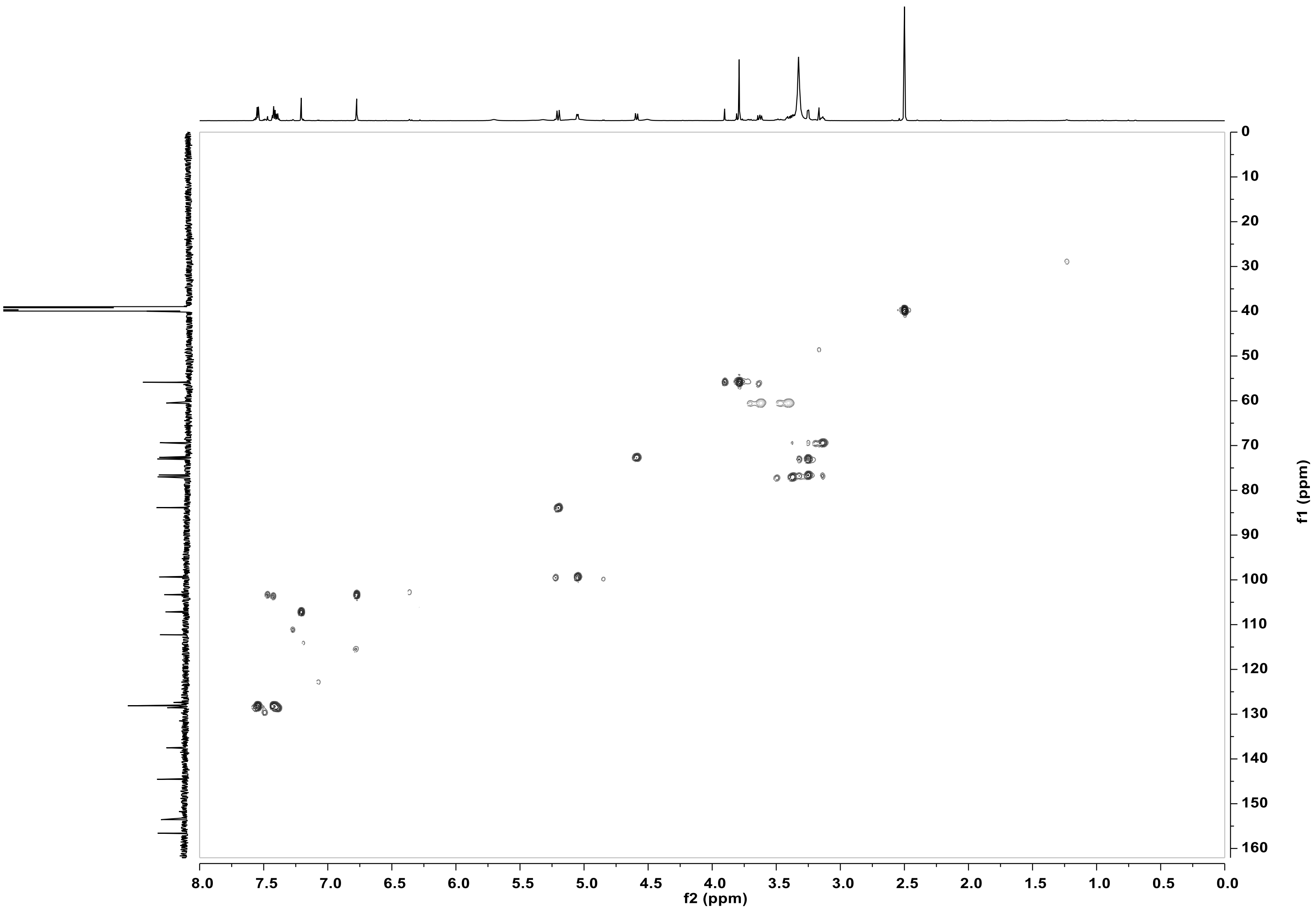

Figure S6. HMQC (700 MHz, DMSO-d_6) spectrum of **2.**

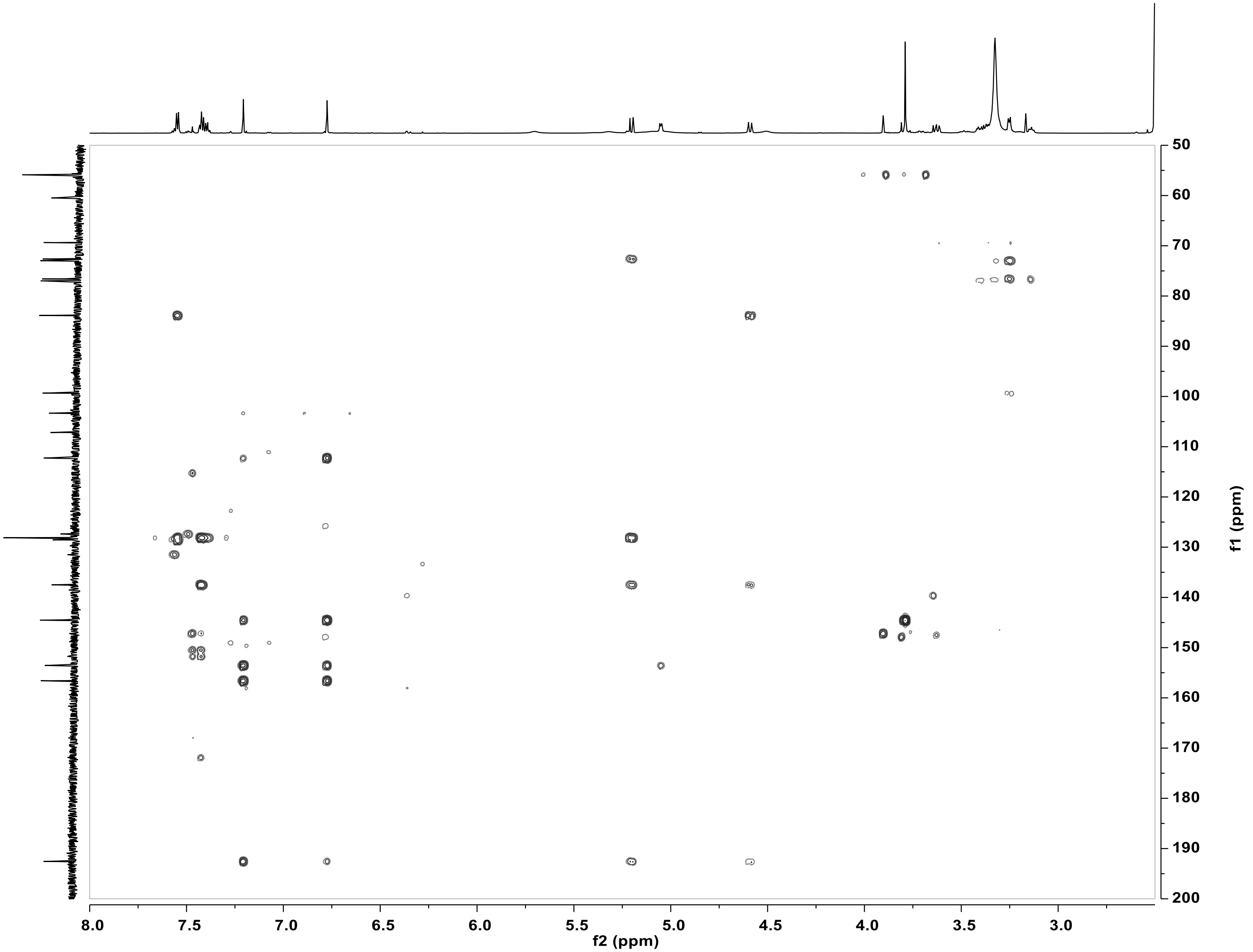

Figure S7. HMBC (700 MHz, DMSO-d_6) spectrum of **2.**

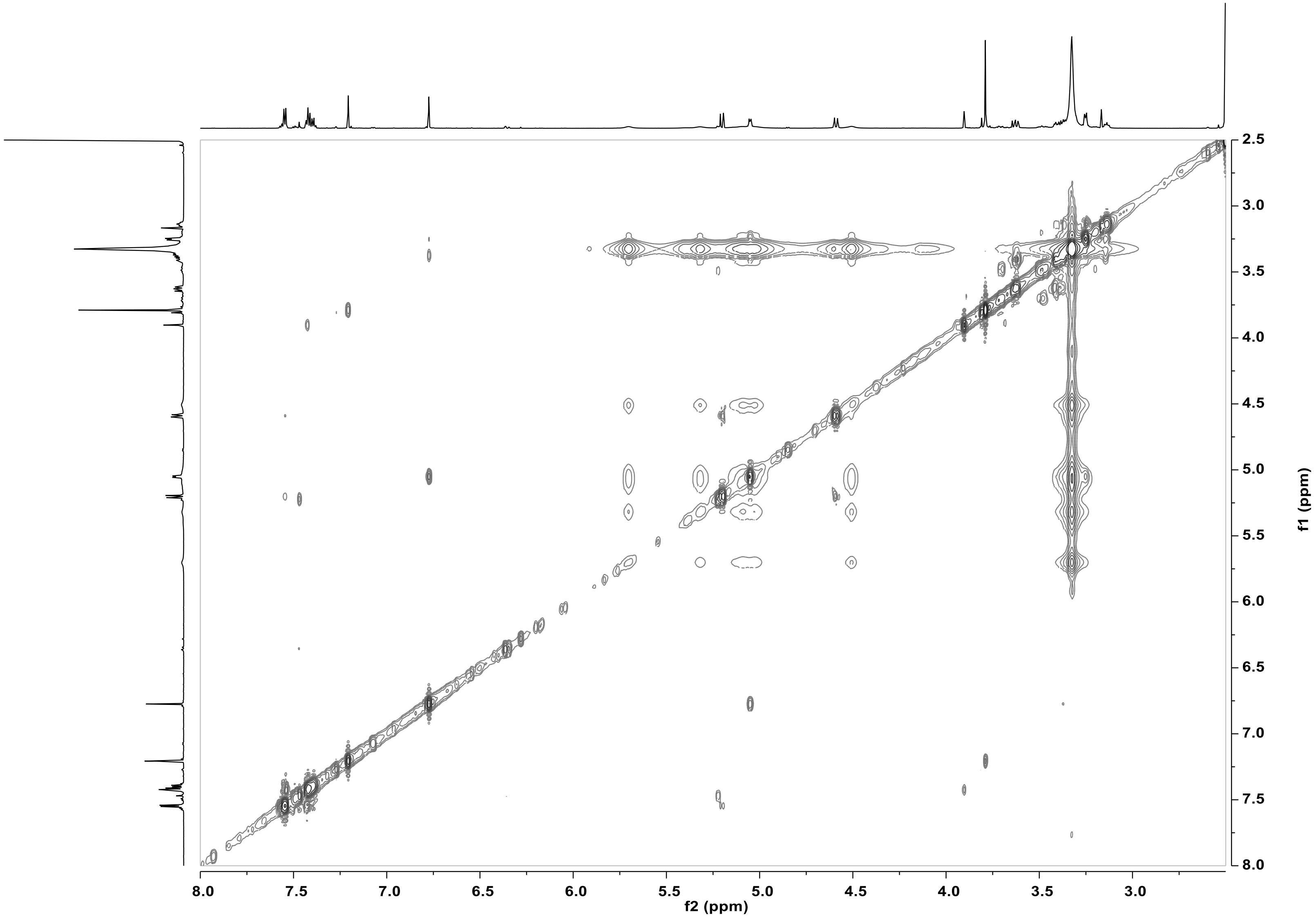

Figure S8. NOESY (500 MHz, DMSO-d_6) spectrum of **2.**

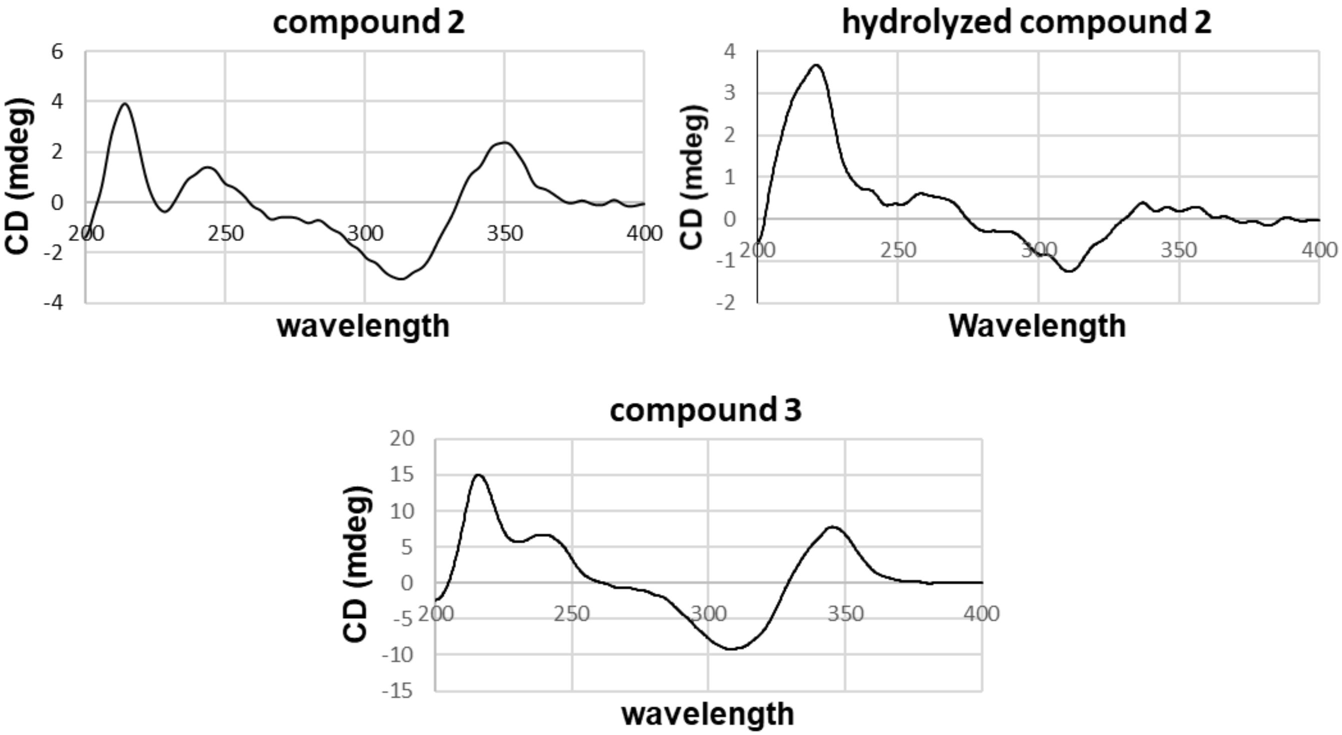

Figure S9. CD spectrum of **2** and **3.**

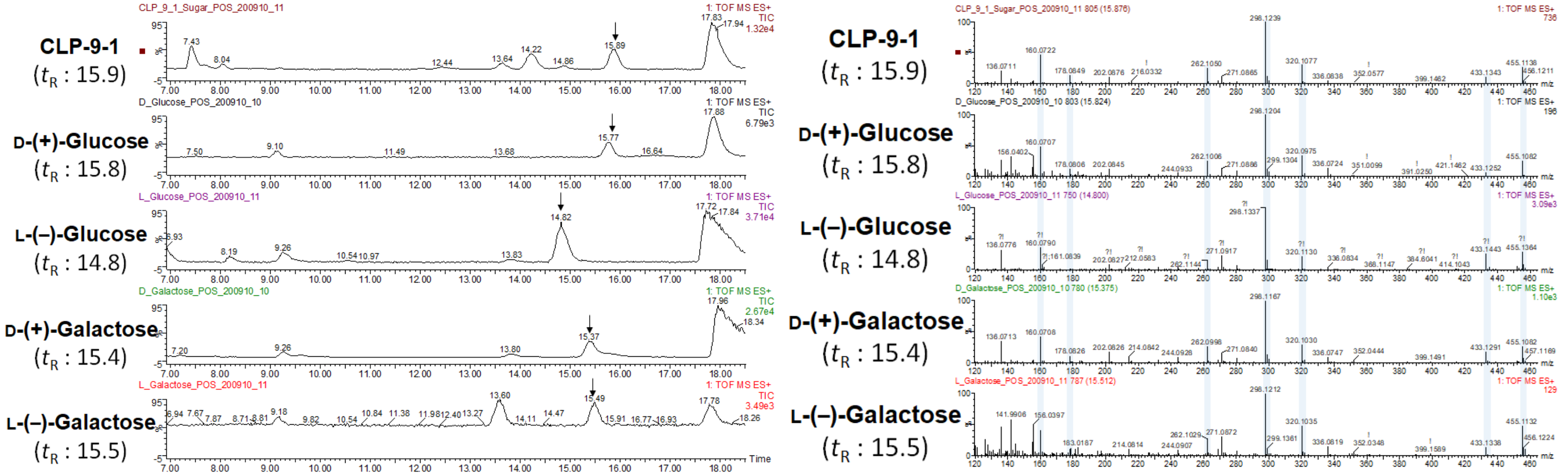

Sub-group	Peak order	Compound name	t_R (min)	Fragment ions (*m/z*)
Hexose				
	1	L-allose	8.7	455, 433, 320, 298, 160, 136
	2	L-glucose	13.3	455, 433, 320, 298, 262, 178, 160, 136
	3	D-galactose	13.9	455, 433, 320, 298, 262, 178, 160, 136
	4	L-galactose	14.4	455, 433, 320, 298, 262, 178, 160, 136
	5	D-glucose	14.7	455, 433, 320, 298, 262, 178, 160, 136
	6	D-allose	15.8	455, 433, 320, 298, 262, 178, 160, 136

Planta Med 2012, 78, 834-837

Figure S10. Determination of sugar contents using UPLC-QTOF-MS.

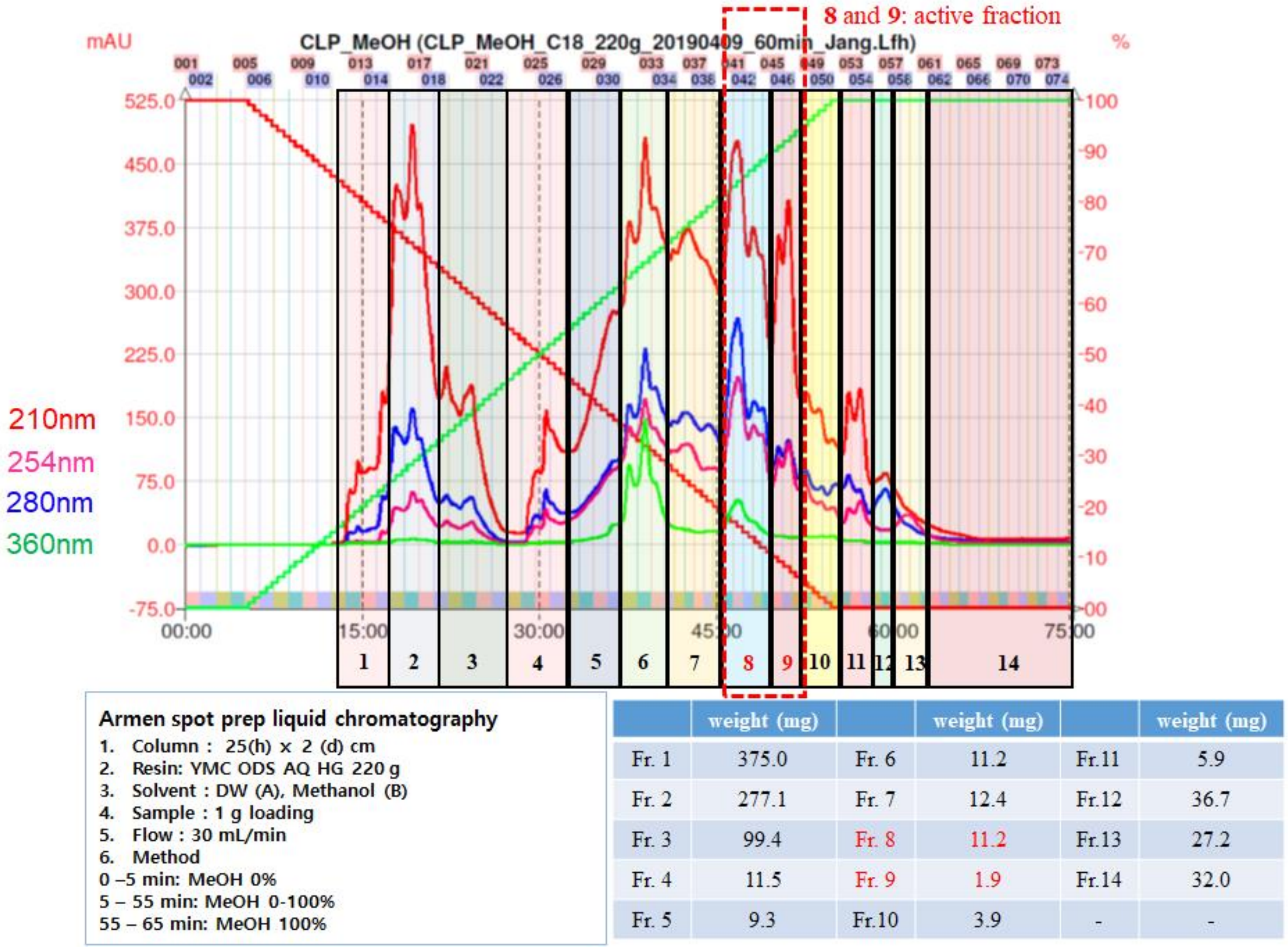

Armen spot prep liquid chromatography

1. Column : 25(h) x 2 (d) cm
2. Resin: YMC ODS AQ HG 220 g
3. Solvent : DW (A), Methanol (B)
4. Sample : 1 g loading
5. Flow : 30 mL/min
6. Method

0 –5 min: MeOH 0%
5 – 55 min: MeOH 0-100%
55 – 65 min: MeOH 100%

	weight (mg)		weight (mg)		weight (mg)
Fr. 1	375.0	Fr. 6	11.2	Fr.11	5.9
Fr. 2	277.1	Fr. 7	12.4	Fr.12	36.7
Fr. 3	99.4	Fr. 8	11.2	Fr.13	27.2
Fr. 4	11.5	Fr. 9	1.9	Fr.14	32.0
Fr. 5	9.3	Fr.10	3.9	-	-

Figure S11. MPLC fractionation of *Canavalia lineata* pod (CLP) MeOH extract for bioactivity-guided separation.

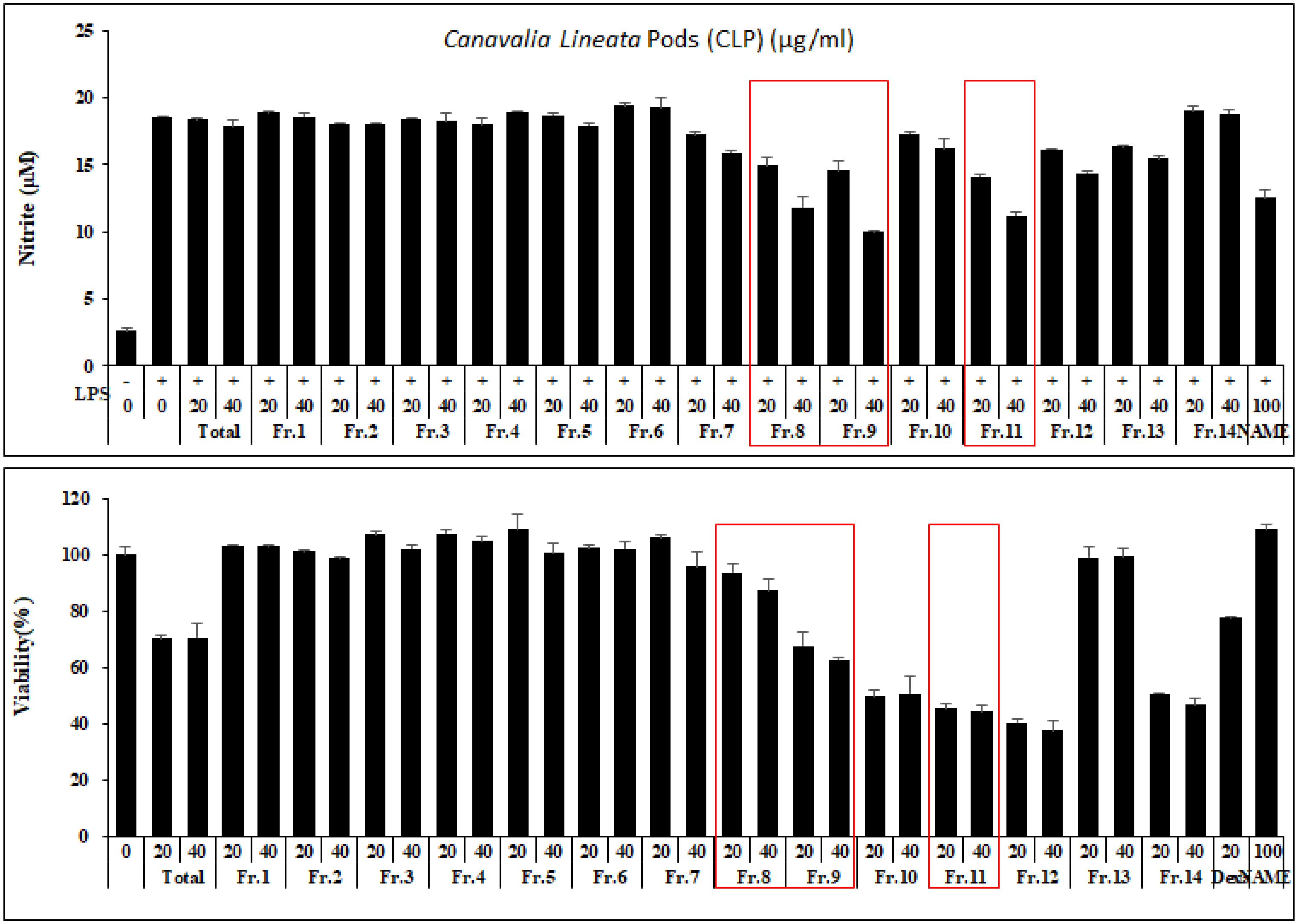

Figure S12. Anti-inflammatory effects of CLP MeOH fractions 1–14.

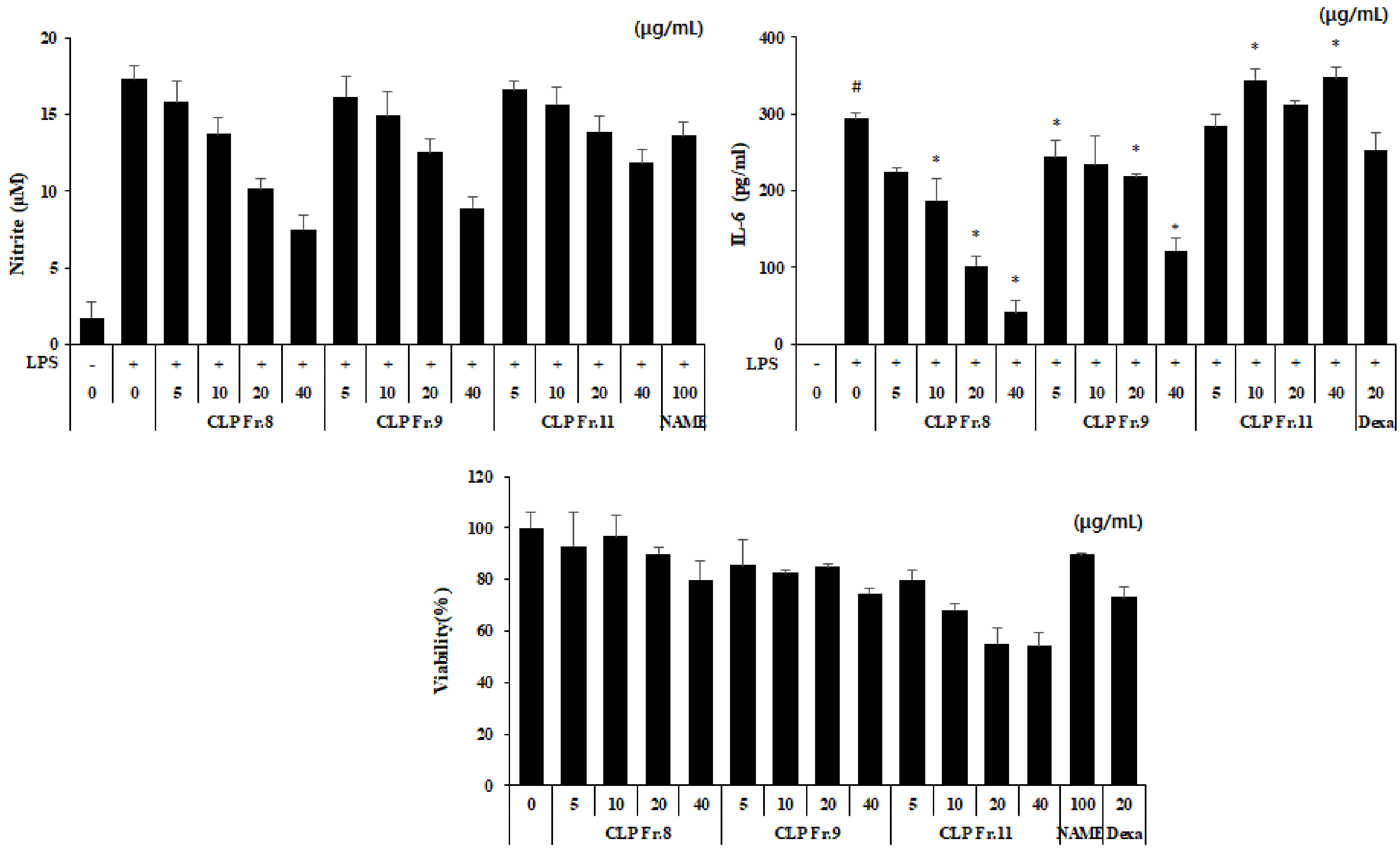

Figure S13. Anti-inflammatory effects of bioactive subfractions 8, 9, and 11.

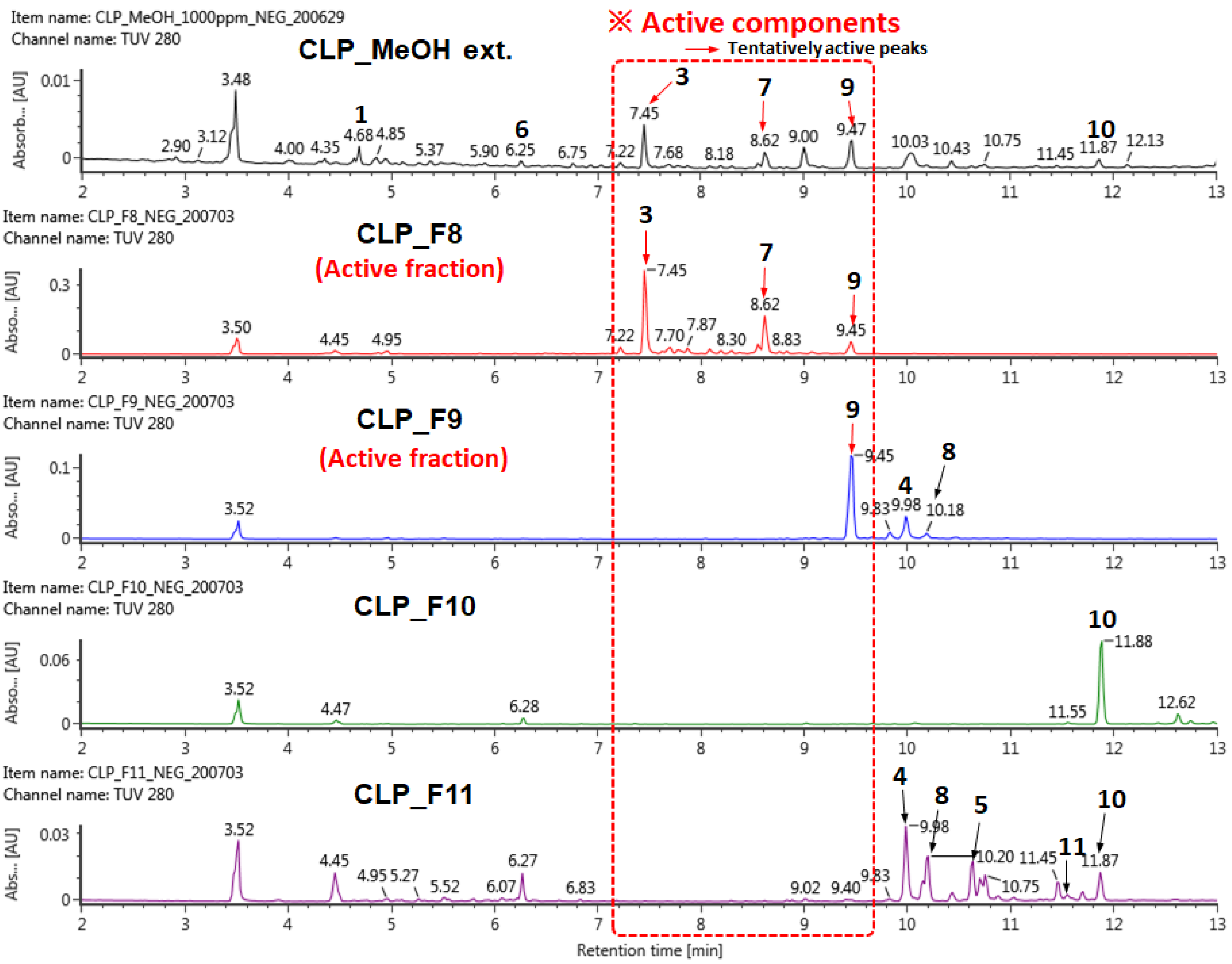

Figure S14. UPLC analysis (280 nm) of bioactive subfractions 8–11.

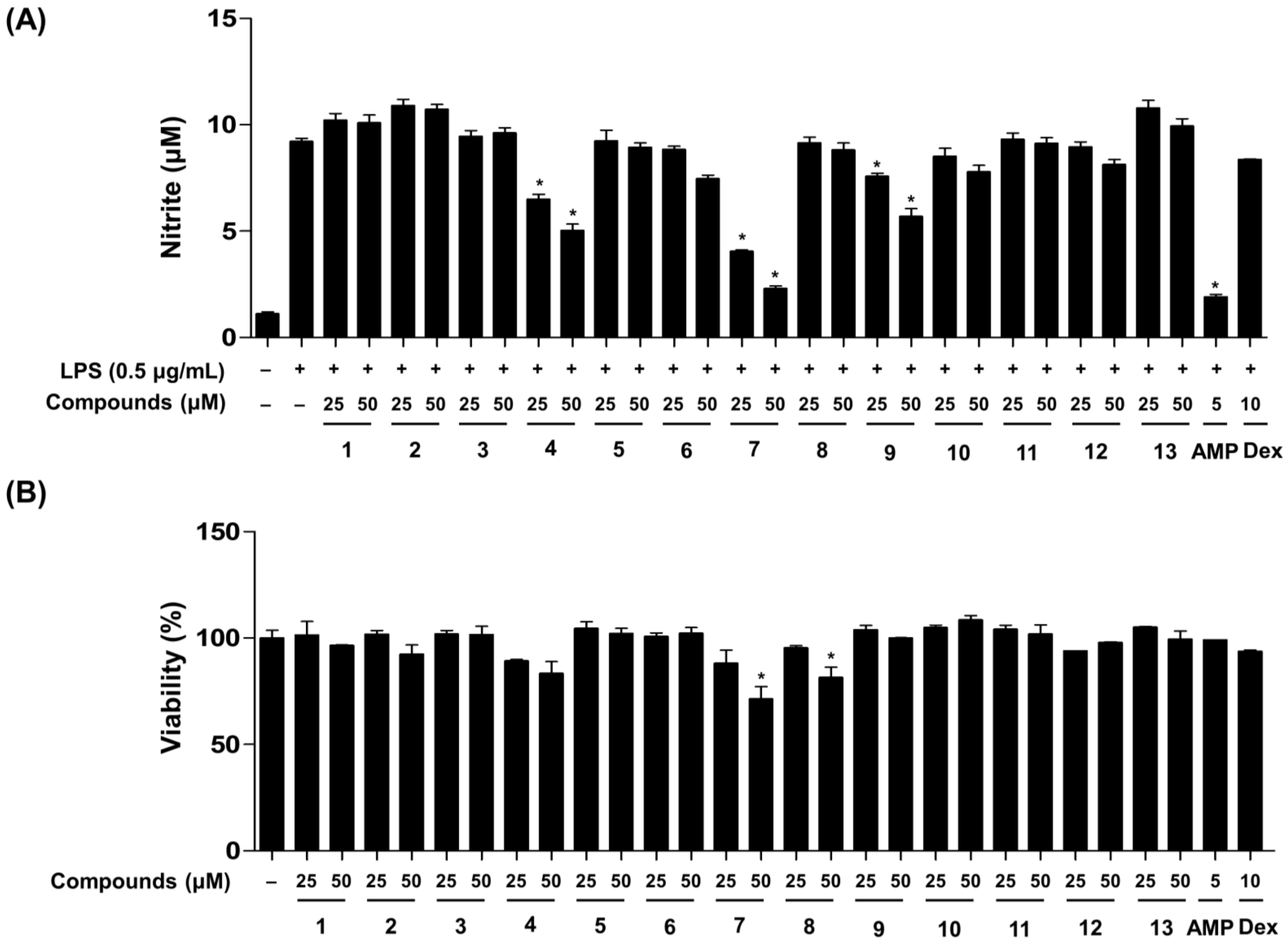

Figure S15. Anti-inflammatory effects of compounds **1**–**13** on LPS-induced NO production.

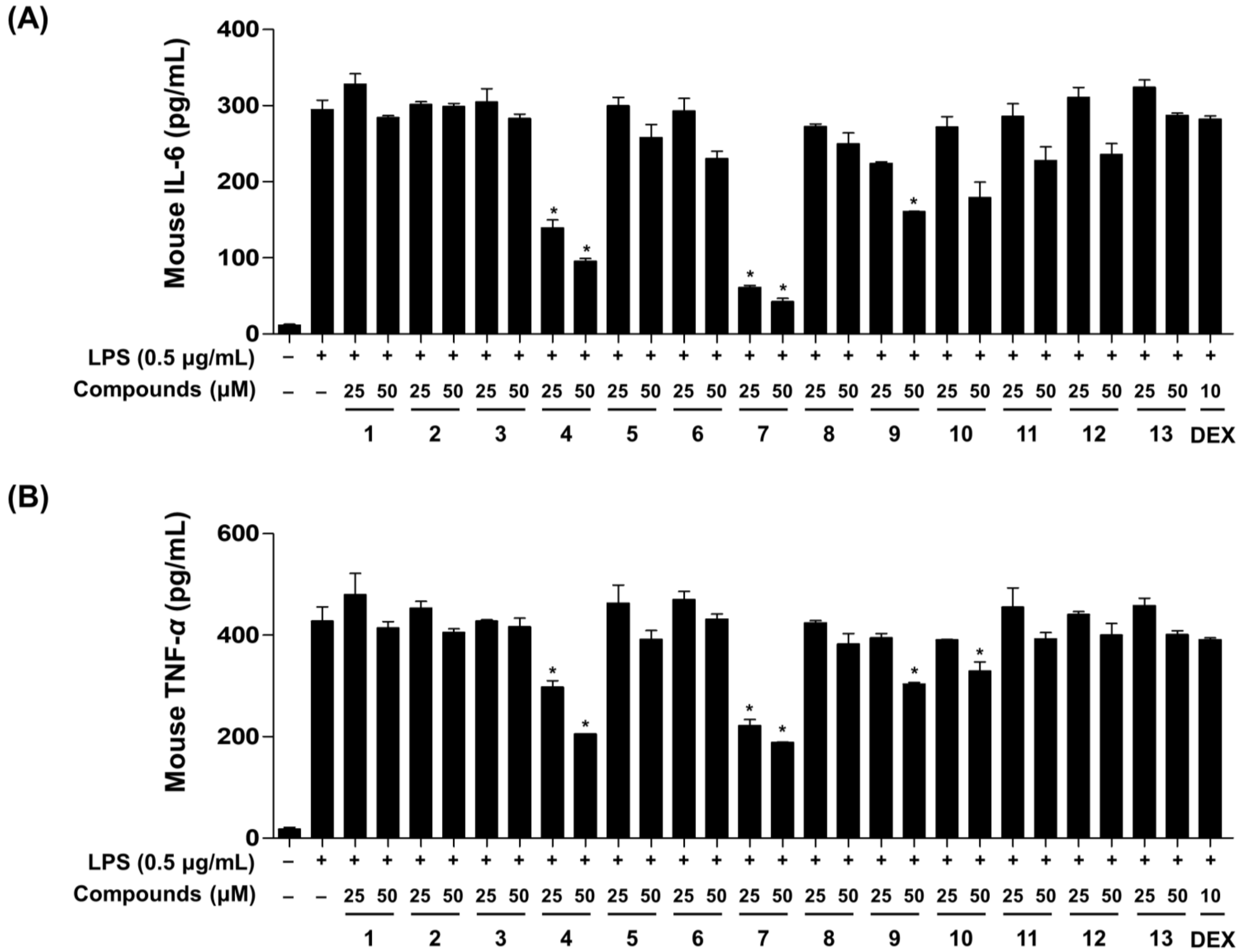

Figure S16. Anti-inflammatory effects of compounds **1**–**13** on LPS-induced IL-6 (A) and TNF-α (B) production.

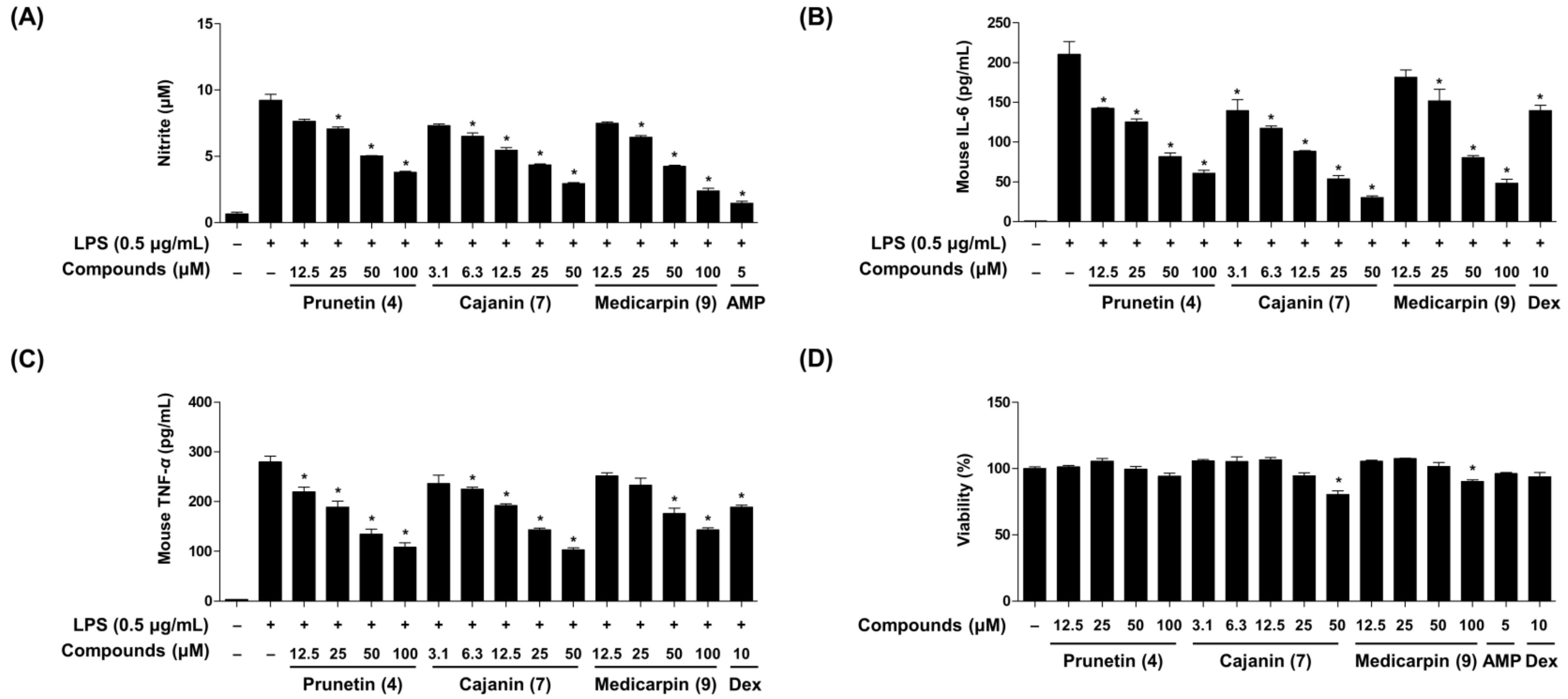

Figure S17. Anti-inflammatory effects of compounds **4**, **9,** and **7** on LPS-induced NO (A), IL-6 (B), and TNF-α (C) production.

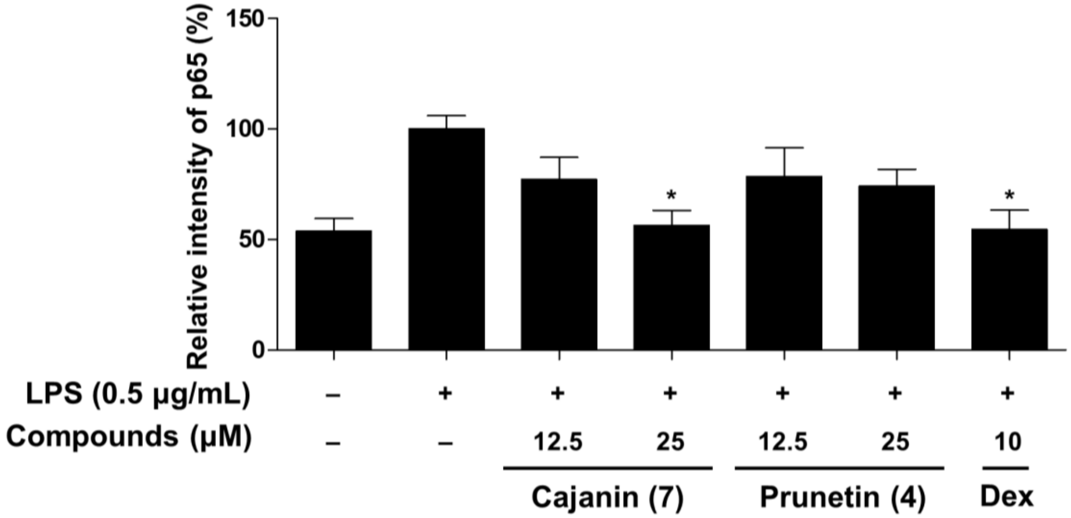

Figure S18. Fluorescence intensity of p65 translocated to the nucleus. The fluorescence intensity of p65 translocated to the nucleus of each cell was calculated by ImageJ software (ver. 1.53a) and all fluorescence intensity values for each groups were normalized to the LPS-only treatment group. Data are indicated as the mean ± SEM of three independent experiments. Significant differences are expressed as asterisks between the LPS-only and experimental groups (*$p < 0.05$).

Table S1. Climate condition for harvesting of *C. lineata*.

Date	Climate conditions [1)						
	Temperature (°C)			Precipitation (mm)	Total sunshine hours (hr)	Humidity (%)	Wind velocity (m/s)
	Average	Min.	Max.				
Jan. 2019	5.8	2.7	9.3	16.2	160.7	63	3.2
Feb. 2019	7.0	3.5	10.7	77.4	139.6	62	3.4
Mar. 2019	10.2	5.5	14.9	133.5	198.7	62	3.5
Apr. 2019	13.9	9.4	18.2	132.5	193.3	69	2.8
May 2019	18.7	13.3	23.6	247.3	268.7	62	3.0
Jun. 2019	20.9	17.5	24.2	170.2	196.8	80	2.6
Jul. 2019	24.1	21.9	26.9	516.5	134.1	87	3.5
Aug. 2019	26.7	23.9	30.2	377.4	212.3	81	2.7
Sep. 2019 [2)]	23.9	21.5	26.7	613.3	144.9	82	3.6
Oct. 2019	19.1	15.5	22.5	233.9	217.0	70	3.5

Plants were cultivated in Gujwa-eup, Jeju-do, Republic of Korea.
Growth conditions of *C. lineata* were cited in Korea Meteorological Administration.
[1)] Meteorological data of the cultivated region were investigated as monthly averages.
[2)] Harvest date is September 5, 2019.
